# Crosstalk between *Neospora caninum* and the bovine host at the maternal-foetal interface determines the outcome of infection

**DOI:** 10.1186/s13567-020-00803-y

**Published:** 2020-06-17

**Authors:** Laura Jiménez-Pelayo, Marta García-Sánchez, Esther Collantes-Fernández, Javier Regidor-Cerrillo, Pilar Horcajo, Daniel Gutiérrez-Expósito, José Espinosa, Julio Benavides, Koldo Osoro, Christiane Pfarrer, Luis Miguel Ortega-Mora

**Affiliations:** 1 Animal Health Department, Faculty of Veterinary Sciences, SALUVET, Complutense University of Madrid, Ciudad Universitaria s/n, 28040 Madrid, Spain; 2 Faculty of Veterinary Sciences, SALUVET-innova, Complutense University of Madrid, Avda. Puerta de Hierro S/N, 28040 Madrid, Spain; 3 Instituto de Ganadería de Montaña (CSIC-Universidad de León), 24346 León, Spain; 4 Regional Service for Research and Agri-Food Development (SERIDA), 33300 Villaviciosa, Asturias, Spain; 5 Department of Anatomy, University of Veterinary Medicine Hannover, Bischofsholer Damm 15, 30173 Hannover, Germany

## Abstract

*Neospora caninum* is an apicomplexan cyst-forming parasite that is considered one of the main causes of abortion. The pathogenic mechanisms associated with parasite virulence at the maternal-foetal interface that are responsible for the outcome of infection are largely unknown. Here, utilizing placentomes from cattle experimentally infected with high-virulence (Nc-Spain7) and low-virulence (Nc-Spain1H) isolates, we studied key elements of the innate and adaptive immune responses, as well as components of the extracellular matrix (ECM), at 10 and 20 days post-infection (dpi). The low-virulence isolate elicited a robust immune response characterized by upregulation of genes involved in pathogen recognition, chemokines and pro-inflammatory cytokines, crucial for its adequate control. In addition, Nc-Spain1H triggered the expression of anti-inflammatory cytokines and other mechanisms implicated in the maintenance of ECM integrity to ensure foetal survival. In contrast, local immune responses were initially (10 dpi) impaired by Nc-Spain7, allowing parasite multiplication. Subsequently (20 dpi), a predominantly pro-inflammatory Th1-based response and an increase in leucocyte infiltration were observed. Moreover, Nc-Spain7-infected placentomes from animals carrying non-viable foetuses exhibited higher expression of the IL-8, TNF-α, iNOS and SERP-1 genes and lower expression of the metalloproteases and their inhibitors than Nc-Spain7-infected placentomes from animals carrying viable foetuses. In addition, profound placental damage characterized by an alteration in the ECM organization in necrotic foci, which could contribute to foetal death, was found. Two different host-parasite interaction patterns were observed at the bovine placenta as representative examples of different evolutionary strategies used by this parasite for transmission to offspring.

## Introduction

*Neospora caninum* is an apicomplexan cyst-forming protozoan parasite that is considered one of the main causes of abortion and one of the organisms most efficiently transmitted by the transplacental route in cattle [1, 2]. The invasion and proliferation of *N. caninum* in the placenta and its dissemination to the foetus are crucial events in the pathogenesis of bovine neosporosis [2, 3]. In addition to its barrier function, the placenta can act as an immunoregulatory organ by recognizing pathogens via pathogen recognition receptors (PRRs), resulting in cytokine production and the regulation of co-stimulatory molecules [4, 5]. However, little is known about the interaction of *N. caninum* with the maternal-foetal interface, particularly at the early stages of infection.

In addition, the factors that enable some isolates to be more effectively transmitted or cause foetal death than others are still unclear. Previously, we used in vitro and in vivo models to characterize two *N. caninum* isolates with marked differences in virulence: Nc-Spain7 and Nc-Spain1H, previously classified as high- and low-virulence isolates, respectively [6–8]. Specifically, in bovine trophobast cells [9–11] and macrophages [12], Nc-Spain7 showed an increased infection and proliferation rates, whereas Nc-Spain1H displayed a reduced proliferation associated to a higher stimulation of immune responses. However, in vitro models cannot mimic the complex architecture of the bovine placenta, as they lack the microenvironmental influences and the host ability to compensate for stress conditions. Recently, we used an in vivo model of bovine infection at mid-gestation to study the early infection dynamics (10 and 20 days post-infection, dpi) after experimental challenge with high- and low-virulence isolates of *N. caninum* (Nc-Spain7 and Nc-Spain1H, respectively) [13]. The results confirmed marked differences in virulence. Specifically, Nc-Spain7 induced foetal death and vertical transmission, with increased dissemination, parasite burdens and lesion severity in placental and foetal tissues. However, the infection with the low-virulence isolate Nc-Spain1H did not result in foetal death and lesional development.

Herein, the interactions of *N. caninum* with the bovine placenta were investigated by comparing the mRNA expression of key elements of the immune response (PRRs, cytokines, chemokines and endothelial adhesion molecules genes), as well as implicated immune cell populations and distribution of components of the extracellular matrix (ECM). The results from this work revealed a differential pattern of response at the placental level after infection with high- and low-virulence isolates. In addition, they may allow us to understand the role of immune responses at the maternal-foetal interface in determining foetal death or survival and congenital transmission.

## Materials and methods

### Animals and experimental design

A full description of the animals and experimental design have been previously published [13]. Briefly, pregnant Asturian heifers (*n* = 24) were randomly distributed into three experimental groups inoculated intravenously at 110 days of gestation with phosphate-buffered saline (PBS G-control; *n* = 6) or 10^7^ culture-derived tachyzoites of the Nc-Spain7 isolate (G-NcSpain7; *n* = 9) or the Nc-Spain1H isolate (G-NcSpain1H; *n* = 9). Three animals from G-control, four animals from G-NcSpain7 and G-NcSpain1H groups, respectively, were culled at 10 dpi. No foetal death was observed at 10 dpi in any of the infected groups, and placental samples from only one Nc-Spain7-infected heifer was *N. caninum*-DNA positive by PCR. Three animals from the G-control group, five animals from the G-NcSpain7 and G-NcSpain1H groups, respectively, were culled at 20 dpi. Foetal death was detected during culling in two heifers from the G-NcSpain7 group. In the G-NcSpain7 group, parasite DNA was detected in 97.8% of the placenta samples analysed and in 100% of the foetuses from animals culled at 20 dpi. In the G-NcSpain1H group, only placental samples were positive in one animal and transmission to the foetus was not demonstrated up to 20 dpi [13].

### Sample collection

Three randomly medial placentomes were carefully detached by hand, and maternal caruncles (CA) and foetal cotyledons (CO) were separated and placed in RNAlater (Sigma-Aldrich, USA) and stored at −80 °C until used for mRNA expression analysis. In addition, non-separated placentomes were transversally cut into slices of 2–3 mm thickness and stored in 10% neutral-buffered formalin or snap frozen and stored at −80 °C (Sigma-Aldrich, USA) for immunohistochemical labelling.

### RNA extraction and reverse transcription

Quantitative real time PCR (qPCR) was used to study the mRNA expression levels of PRRs (TLR-2, TLR-3, TLR-8, TLR-9 and NOD2), pro-inflammatory cytokines (IL-1β, IL-6, IL-8, IL-12p40, IL-17A, IFN-γ, TNF-α), anti-inflammatory/regulatory cytokines (IL-4, IL-10, and TGF-β1), the cytokine-inducible nitric oxide synthase (iNOS), chemokines (CCL2, CCL4, and CCL5), endothelial adhesion molecules (ICAM-1 and VCAM-1) and genes related to ECM remodelling (the metalloproteases MMP-2, MMP-13, MMP-14; their inhibitors TIMP-1, TIMP-2; and SERP-1). Total RNA was extracted from approximately 10 mg of three medial CAs and COs from each animal using a commercial Maxwell^®^ 16 LEV simplyRNA Purification Kit, developed for the automated Maxwell^®^ 16 System (Promega, USA), following the manufacturer’s recommendations. Reverse transcription was carried out by the master mix SuperScript^®^ VILO™ cDNA Synthesis Kit (Invitrogen, UK) in 20 μL reactions using up to 2.5 μg of total RNA. The obtained cDNA products were diluted 1:20 in molecular-grade water and used in qPCR assays.

### Quantitative real-time PCR

qPCR reactions were performed using 12.5 µL of Power SYBR^®^ Green PCR Master Mix (Applied Biosystems, USA), 10 pmol of each primer and 5 μL of diluted cDNA samples on an ABI 7500 Fast Real-Time PCR System (Applied Biosystems, USA). Primers for the qPCRs are shown in Additional file 1. The β-Actin and GAPDH were used as housekeeping genes. For each target gene, a seven-point standard curve was included in each batch of amplifications based on tenfold serial dilutions starting at 10 ng/µL plasmid DNA or PCR product. The relative quantification of the mRNA expression levels (x-fold change in expression) was carried out by the comparative 2–ΔΔCt method [14].

### Immunohistochemistry

The presence and distribution of *N. caninum* antigens, T lymphocytes (CD3 + , CD4 + and CD8 + cell populations), B lymphocytes (CD20 +), macrophages (MAC387 + and lysozyme +), iNOS staining, MMPs (MMP-2 and MMP-14), TIMP-1 and ECM components (laminin, fibronectin and collagen type IV) were analysed by immunohistochemistry (IHC) in three randomly selected medial placentomes. The primary antibodies and immunostaining protocols used are listed in Additional file 2. Samples fixed in 10% neutral-buffered formalin were dehydrated with a graded series of alcohol solutions and embedded in paraffin wax for histopathological and immunohistochemical studies. The samples were subjected to different pre-treatments according to the recommendations of the specific antibody manufacturer to expose the antigen of interest. In all cases, a polymer-based detection system (EnVision^®^ System Labelled Polymer-HRP; Dako, Denmark) was employed, and immunolabelling was developed with a solution of 3,3′diaminobenzidine (DAB; Vector Laboratories, USA). The slides were counterstained with Harris haematoxylin and mounted in hydrophobic medium EuKitt^®^ (Sigma-Aldrich, USA). In the specific case of T cell subpopulation characterization, CD4 + and CD8 + antigens were immunolabelled on cryostat sections from snap frozen samples. Steps followed for the immunohistochemical labelling were the same as those for paraffin sections except for deparaffination and antigen retrieval, which are not necessary for cryostat sections.

### Counting or scoring the positive infiltration

T lymphocytes (CD3 + , CD4 + and CD8 + cell populations), B lymphocytes (CD20 +) and phagocytic cells (MAC387 + and/or lysozyme +) were quantified in the three selected placentomes per animal using light microscopy (Leica ICC50W, Leica Microsystems, Germany). The number of labelled cells was counted in 20 random 20 × fields within the endometrial chorioallantoic interdigitation area of the placentome using ImageJ software. In contrast, the presence and distribution of *N. caninum* antigens, iNOS staining and the labelling of MMPs (MMP-2 and MMP-14), TIMP-1 and ECM components (laminin, fibronectin and collagen type IV) was evaluated subjectively in the same placentomes due to their diffuse distribution. Immunolabelling was defined as the brownish colour present in the cytosolic compartment of the cells and in the ECM. A descriptive analysis of the immunolabelling was performed, and the immunolabelling pattern of MMPs, TIMPs and ECM components was semi-quantitatively analysed using the following scale values: −no immunolabelling (total absence of immunoreactive cells); + low immunolabelling; ++ moderate immunolabelling; +++ strong immunolabelling; and ++++ extremely strong immunolabelling. To eliminate inter-operator error, all slides were read by a single investigator.

### Statistical analysis

Messenger RNA expression levels and the number of CD3 + , CD4 + , CD8 + , CD20 + , MAC387 + and lysozyme-positive cells in the placenta were analysed using the non-parametric Kruskal–Wallis test, followed by a Dunn’s multiple range test to compare groups. In addition, to assess differences between infected groups and between animals carrying non-viable foetuses (NVFs) and viable foetuses (VFs) within the G-NcSpain7 group culled at 20 dpi, a Mann–Whitney test was performed. Finally, a non-parametric Spearman correlation test was performed to study whether increases in immune cell populations in the placenta were associated with an increase in cytokine expression. The statistical significance for all the analyses was established as *P* < 0.05. GraphPad Prism 5 v.5.01 (San Diego, USA) software was used to perform all the statistical analyses and create all the graphical illustrations.

## Results

### The parasite isolate determined a time-dependent expression pattern of PRRs chemokines and endothelial adhesion molecule genes in placentomes

**Figure 1:**
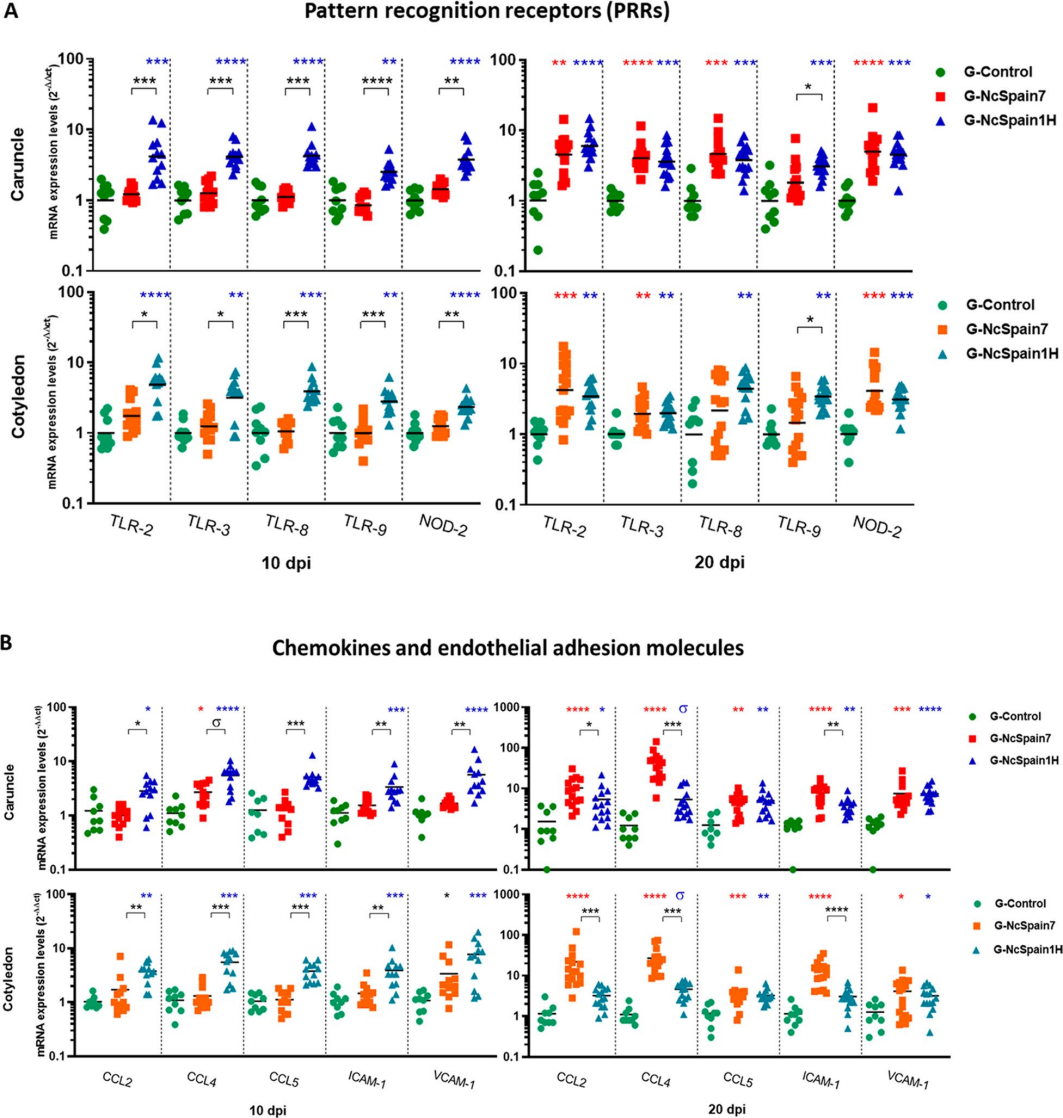

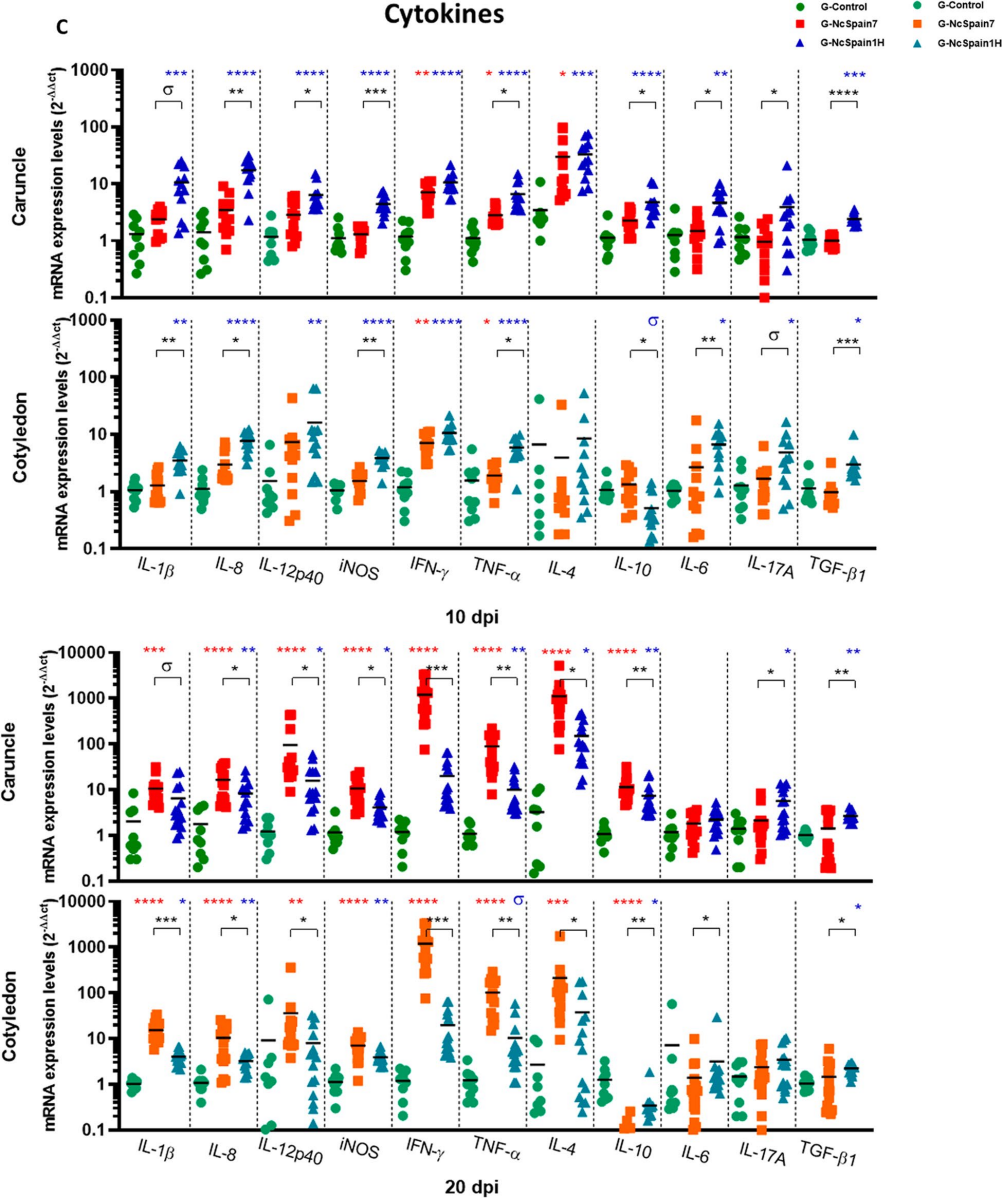

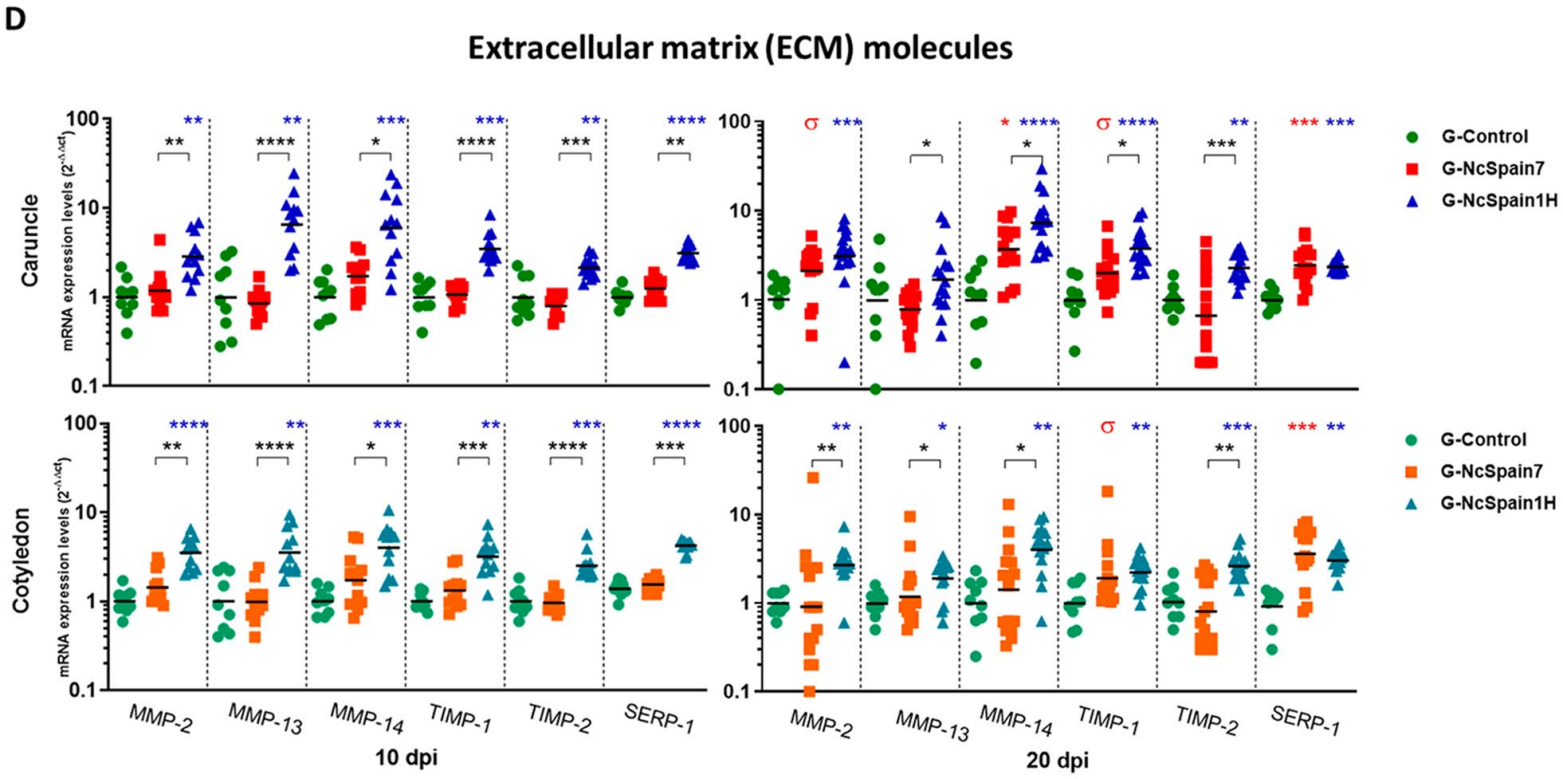
**PRRs, cytokine, chemokine, endothelial adhesion molecules, MMP and TIMP transcript expression.** Scatter-plot graphs of relative mRNA expression levels (as the fold change) of PRRs **(A)**, cytokines **(B)**, chemokines and endothelial adhesion molecules **(C)** and MMPs and TIMPs **(D)** in CA and CO collected at 10 and 20 dpi from cows experimentally infected with Nc-Spain7 and Nc-Spain1H isolates at 110 days of gestation. Data are presented as individual points. Horizontal lines represent median values for each group. ****, ***, ** and * indicate *P* < 0.0001, *P* < 0.001, *P* < 0.01 and *P* < 0.05 significant differences, respectively, and σ indicates *P* < 0.1. Upper symbols represent differences with respect to the control group, and lower symbols represent differences between isolates. Three randomly selected medial caruncles and cotyledons from each animal were analysed.

We found that the expression of all investigated PRRs (Figure  1A) was upregulated in the placentas infected by the low-virulence isolate Nc-Spain1H (*P* < 0.05–0.0001) at 10 and 20 dpi. In contrast, the highly virulent Nc-Spain7 induced RNA expression only at day 20 dpi for almost every PRRs. TLR-9 RNA in both maternal and foetal placenta and TLR-8 in foetal placenta were not over-expressed at any time after Nc-Spain7 infection. A similar pattern was observed for chemokines and endothelial adhesion molecules, being upregulated by the low-virulence isolate at 10 dpi (*P* < 0.05–0.0001), whereas Nc-Spain7 generally failed to significantly induce the expression of any of these molecules (Figure  1B). In general, both isolates also induced the upregulation of CCLs, ICAM-1 and VCAM-1 RNA in placental tissues at the late stages of infection (20 dpi); however, placentomes from animals infected with Nc-Spain7 exhibited higher CCL2, CCL4 and ICAM-1 expression levels than those infected with Nc-Spain1H (*P* < 0.05–0.001), especially in CO samples (Figure  1B).

### Pro-inflammatory cytokines were early induced by Nc-Spain1H and drastically upregulated by Nc-Spain7 at late stage of infection

Infection by the low-virulence isolate Nc-Spain1H triggered an increase in the expression of important pro-inflammatory cytokines such as IL-1β, IL-8, IL-12p40, IFN-γ, TNF-α and iNOS as early as 10 dpi (*P* < 0.1–0.001). However, IFN-γ (*P* < 0.01) and TNF-α (*P* < 0.05) were the only cytokines upregulated by Nc-Spain7 at this time point but with lower mRNA levels than Nc-Spain1H (Figure  1C). Later, at 20 dpi, Nc-Spain1H maintained the RNA levels of pro-inflammatory cytokine expression (*P* < 0.05–0.001) whereas the infection by Nc-Spain7 induced a drastic expression of IFN-γ (1000-fold mean); followed by TNF-α (100-fold mean), IL-12p40 (30-fold mean), IL-1β, IL-8, and iNOS (tenfold mean; *P* < 0.001–0.0001) (Figure  1C). Notably, IFN-γ expression was 100-fold higher in Nc-Spain7-infected placentas than Nc-Spain1H-infected placentas at 20 dpi. On the other hand, Nc-Spain7 infection did not upregulate TGF-β1, IL-6 and IL-17A expression in bovine placentas at any time assayed (Figure  1C). However, we observed an increase in their mRNA levels in placental tissues infected with Nc-Spain1H at 10 dpi (*P* < 0.05–0.001), which remained slightly increased at 20 dpi (*P* < 0.05–0.01).

### High- and low- virulence isolates induced different IL-4 and IL-10 expression levels and Th1/Th2 balances in the caruncle and the cotyledon

IL-4 and IL-10 are key regulators of the immune response and downregulate Th1 response-driven inflammatory reactions, protecting the host from infection-associated immunopathology to maintain placental homeostasis. Regulation of both IL-4 and IL-10 expression varied in maternal and foetal placenta (Figure  1C). The expression of both IL-4 and IL-10 were higher in CA than in CO. The expression of IL-4 was up-regulated in CA infected with both isolates at 10 dpi (*P* < 0.05–0.001) and 20 dpi (*P* < 0.05–0.0001) and in CO infected with Nc-Spain7 at 20 dpi (*P* < 0.001). In the case of IL-10 in CA, higher expression was induced by Nc-Spain1H infection at 10 dpi (*P* < 0.0001) and by both isolates at 20 dpi (*P* < 0.01–0.0001). However, IL-10 expression was down-regulated in COs infected with both isolates at 20 dpi (*P* < 0.05–0.0001) (Figure  1C).

Next, the Th1/Th2 balance was studied as the relation between the IFN-γ/IL-4, to provide information with respect to the predominant microenvironment. The Nc-Spain1H group was not different from the control group at any time in the CA or CO suggesting a Th1/Th2 balance. The Nc-Spain7 isolate induced a predominant Th2 response at 10 dpi in the CAs (*P* < 0.05) that was transformed to a Th1 imbalance in CA and CO at 20 dpi (Additional file 3).

### ECM modulation was induced by Nc-Spain1H infection in bovine placenta

The expression of the better-known MMPs and TIMPs involved in bovine placenta remodelling was increased in the Nc-Spain1H infected placenta samples at 10 and 20 dpi (*P* < 0.01–0.0001) (Figure  1D). MMP and TIMP expression was not regulated by Nc-Spain7 infection at any time-point (Figure  1D). Infection with Nc-Spain1H also upregulated expression of SERP-1 at 10 dpi (Figure  1D), that was maintained at 20 dpi. However, Nc-Spain7 only upregulated SERP-1 expression at 20 dpi (*P* < 0.01–0.0001).

**Figure 2:**
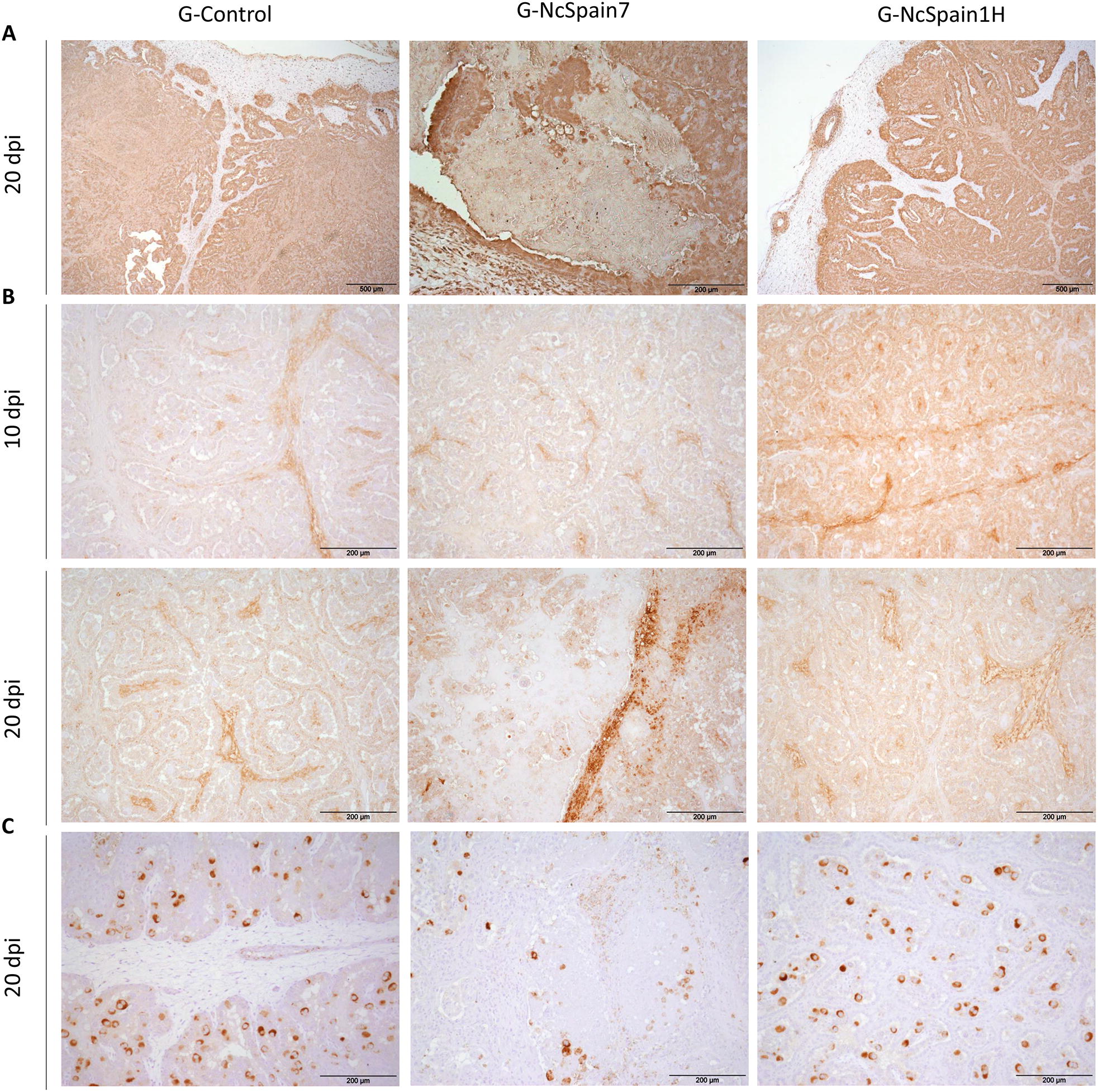
**Comparison of the immunohistochemical labelling of MMP, TIMPs and ECM components**. The distribution of MMP-2 **(A)**, MMP-14 **(B)** and TIMP-2 **(C)** is shown in placentomes from uninfected animals (G-control) and Nc-Spain7- and Nc-Spain1H-infected animals at 10 and 20 dpi. Increased immunoreactivity of MMP-14 was detected in Nc-Spain1H-infected placentomes at 10 dpi. At 20 dpi, a loss of MMP-2, MMP-14 and TIMP-2 staining was observed in the lytic areas of the Nc-Spain7-infected placentomes. Brownish staining indicates a positive reaction. For the magnification, see the bar in each individual image. Three randomly selected medial placentomes from each animal were analysed.

**Figure 3:**
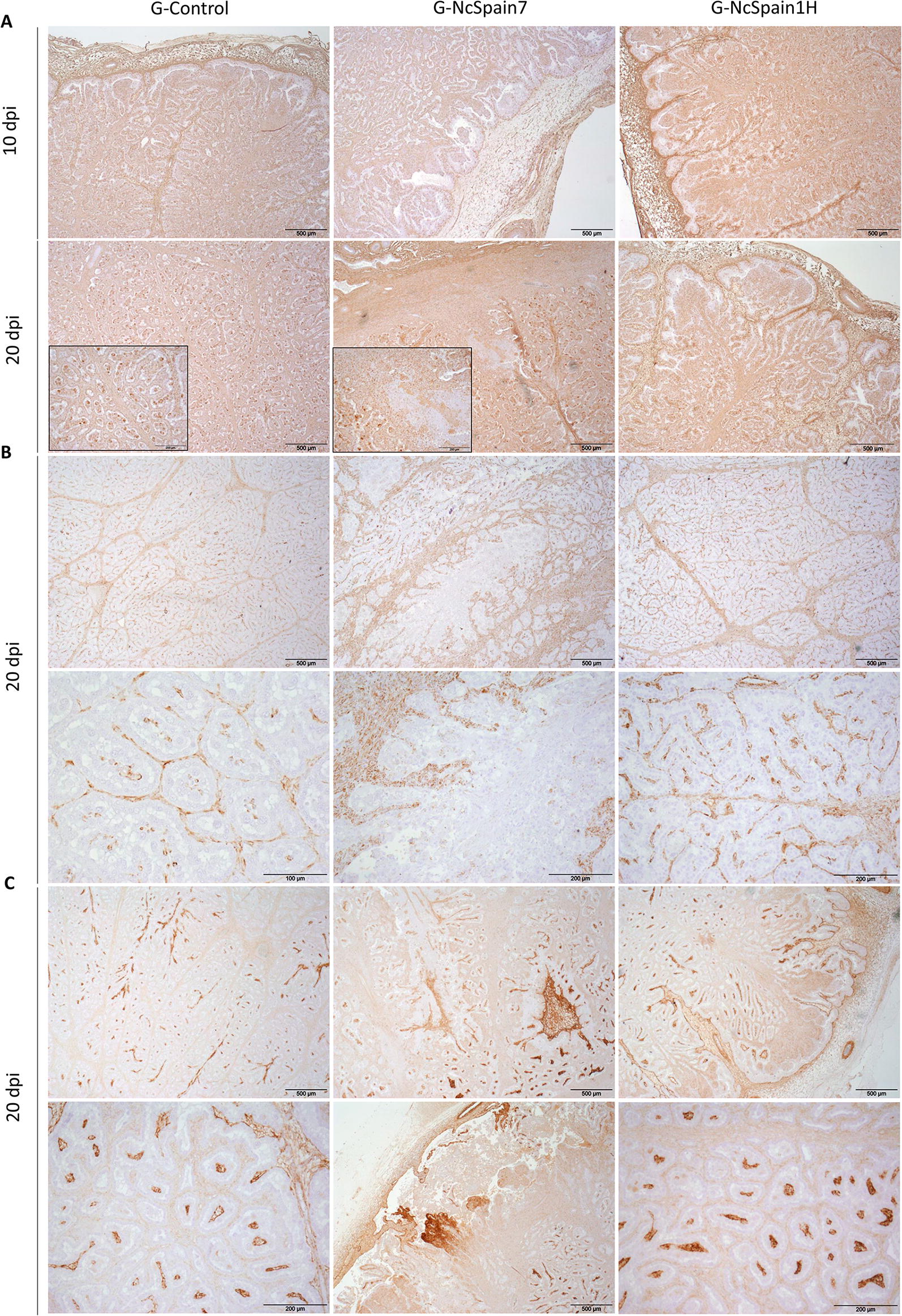
**Comparison of the immunohistochemical labelling of ECM components**. The distribution of fibronectin **(A)**, vimentin **(B)** and collagen IV **(C)** is shown in placentomes from uninfected control animals (G-control) and Nc-Spain7- and Nc-Spain1H-infected animals at 10 and 20 dpi. Increased immunoreactivity of fibronectin was detected in Nc-Spain1H-infected placentomes at 10 dpi. At 20 dpi, a loss of fibronectin, vimentin and collagen IV staining was observed in the lytic areas of the Nc-Spain7-infected placentomes. Brownish staining indicates a positive reaction. For the magnification, see the bar in each individual image. Three randomly selected medial placentomes from each animal were analysed.

The presence and distribution of ECM components in the bovine placenta was studied by IHC (Table 1). We observed an increase in the MMP-14 staining in the maternal stroma and caruncular epithelium of Nc-Spain1H placentomes at 10 dpi (Figure  2B), whereas MMP-2 and TIMP-2 staining was not apparently modified by the infection (not shown). At 20 dpi, MMP-2, MMP-14 and TIMP-2 detection varied relative to the damaged areas, which were present in the Nc-Spain7 infected tissues and were not evenly distributed throughout the whole placentome. Definitively, MMP-2, MMP-14 and TIMP-2 staining was lost in necrotic areas, whereas accumulation of MMP-2 and MMP-14 was found in the areas adjacent to the lesions (Figure  2A–C). In contrast, fibronectin, vimentin and collagen type IV were also investigated as a part of the ECM components susceptible to parasite infection. At 10 dpi, only moderately stronger staining of fibronectin was observed in placentomes infected with Nc-Spain1H (Figure  3A). At 20 dpi, the expression of fibronectin, vimentin and collagen type IV was reduced or absent in the necrotic areas of Nc-Spain7-infected placentomes (Figure  3A–C). In addition, collagen type IV staining was increased at the vicinity of the necrotic areas, although this increase was variable depending on the animal (Figure  3C).

**Table 1:** **Presence and distribution of matrix metalloproteinases (MMPs), inhibitory factors of metalloproteinases (TIMPs) and some components of the extracellular matrix (ECM) in placental samples obtained from an experimental infection model in pregnant cattle**

	Maternal stroma		Caruncular epithelium		Foetal mesenchyme		Uninucleated trophoblast cells		Binucleated trophoblast cells	
	10 dpi	20 dpi	10 dpi	20 dpi	10 dpi	20 dpi	10 dpi	20 dpi	10 dpi	20 dpi
MMP-2										
G-Control	+	+	++	++	–	–	+	+	–	–
G-NcSpain7	+	++	++	+++	–	+	+	++	–	–
G-NcSpain1H	+	+	++		–	+	+	+	–	–
MMP-14										
G-Control	–	+	–	+	++	++	–	+	–	–
G-NcSpain7	–	++	–	+	++	++++	–	+	–	–
G-NcSpain1H	+	+	+	+	++	++	–	+	–	–
TIMP-2										
G-Control	–	–	–	–	–	–	–	–	++++	++++
G-NcSpain7	–	–	–	–	–	–	–	–	++++	++++*
G-NcSpain1H	–	–	–	–	–	–	–	–	++++	++++
Fibronectin										
G-Control	+	+	–	–	++	++	–	–	++	++
G-NcSpain7	+	+/−*	–	–	++	+/−*	–	–	+	+/− *
G-NcSpain1H	++	+	–	–	+++	++	–	–	++	++
Vimentin										
G-Control	+++	+++	–	–	++	++	–	–	–	–
G-NcSpain7	+++	+++/− *	–	–	++	++/− *	–	–	–	–
G-NcSpain1H	+++	+++	–	–	++	++	–	–	–	–
Collagen IV										
G-Control	+	+	–	–	++++	++++	–	–	–	–
G-NcSpain7	+	+/− *	–	–	++++	++++/−*	–	–	–	–
G-NcSpain1H	+	+	–	–	++++	++++	–	–	–	–

− no immunolabeling (total absence of immunoreactive cells); + low immunolabeling; ++ moderate immunolabeling; +++ high immunolabeling; and ++++ extremely high immunolabeling

*Loss of staining in necrotic/damaged areas

### Detection and distribution of *N. caninum* antigens

**Figure 4:**
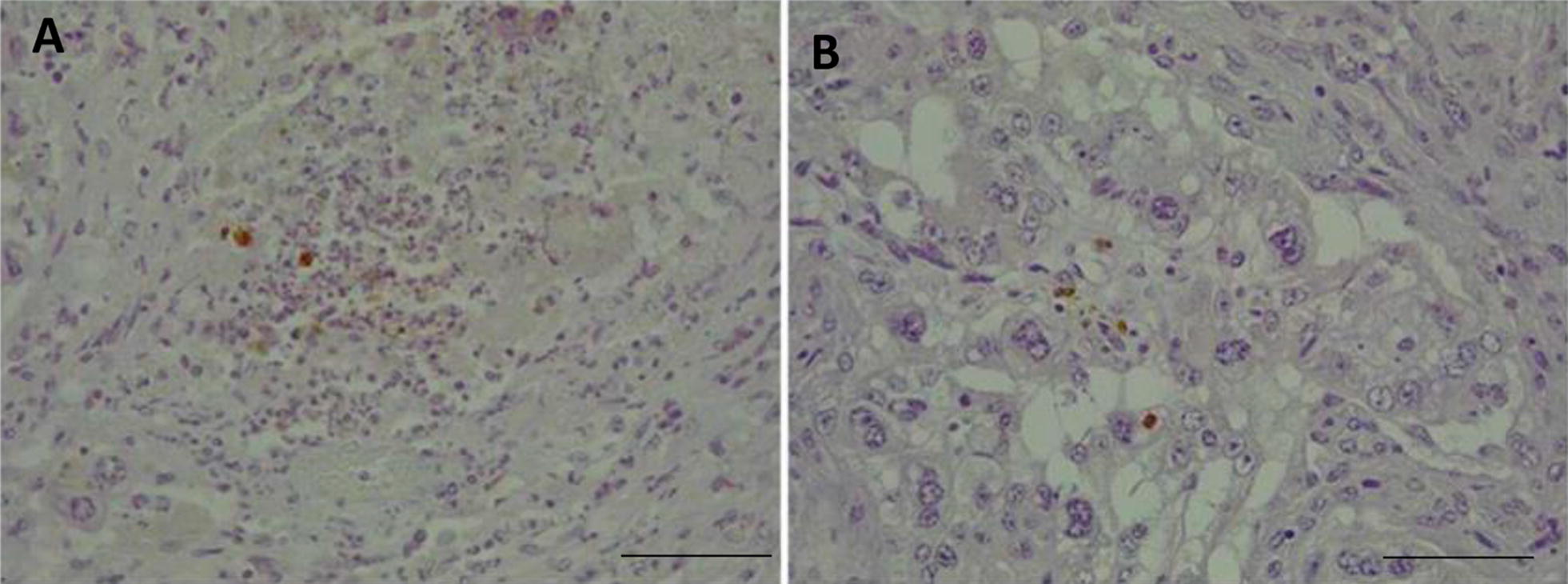
**Immunohistochemical labelling of parasite antigen in the placenta**. *N. caninum* antigen immunohistochemically labelled in placentomes from the G-NcSpain7 group at 20 dpi. Brownish staining indicates a positive reaction. **(A)** Focal necrosis in the caruncular part of the placenta with labelled Nc-Spain7 antigens within trophoblast cells; HE, 10 × , bar 200 µm. **(B)** Labelled Nc-Spain7 antigens within trophoblast cells; HE, 40 × , bar 50 µm. Three randomly selected medial placentomes from each animal were analysed.

The staining of the placentome slides with rabbit-α-*N. caninum* showed the presence of particulate parasite antigens (positively labelled amorphous granular debris), individual tachyzoites, tachyzoite pairs and parasitophorous vacuoles with 4 tachyzoites, localized mainly in the foetal villi of the animals infected with the highly virulent isolate Nc-Spain7 and culled at 20 dpi (Figure  4A and B). *Neospora* antigens were not observed in Nc-Spain1H-infected placentomes by IHC, consistent with PCR detection results [13]. The location of *N. caninum* antigens in relation to the inflammatory cells is described below.

### The high-virulence isolate induced the greatest increase in T and B lymphocytes

**Figure 5:**
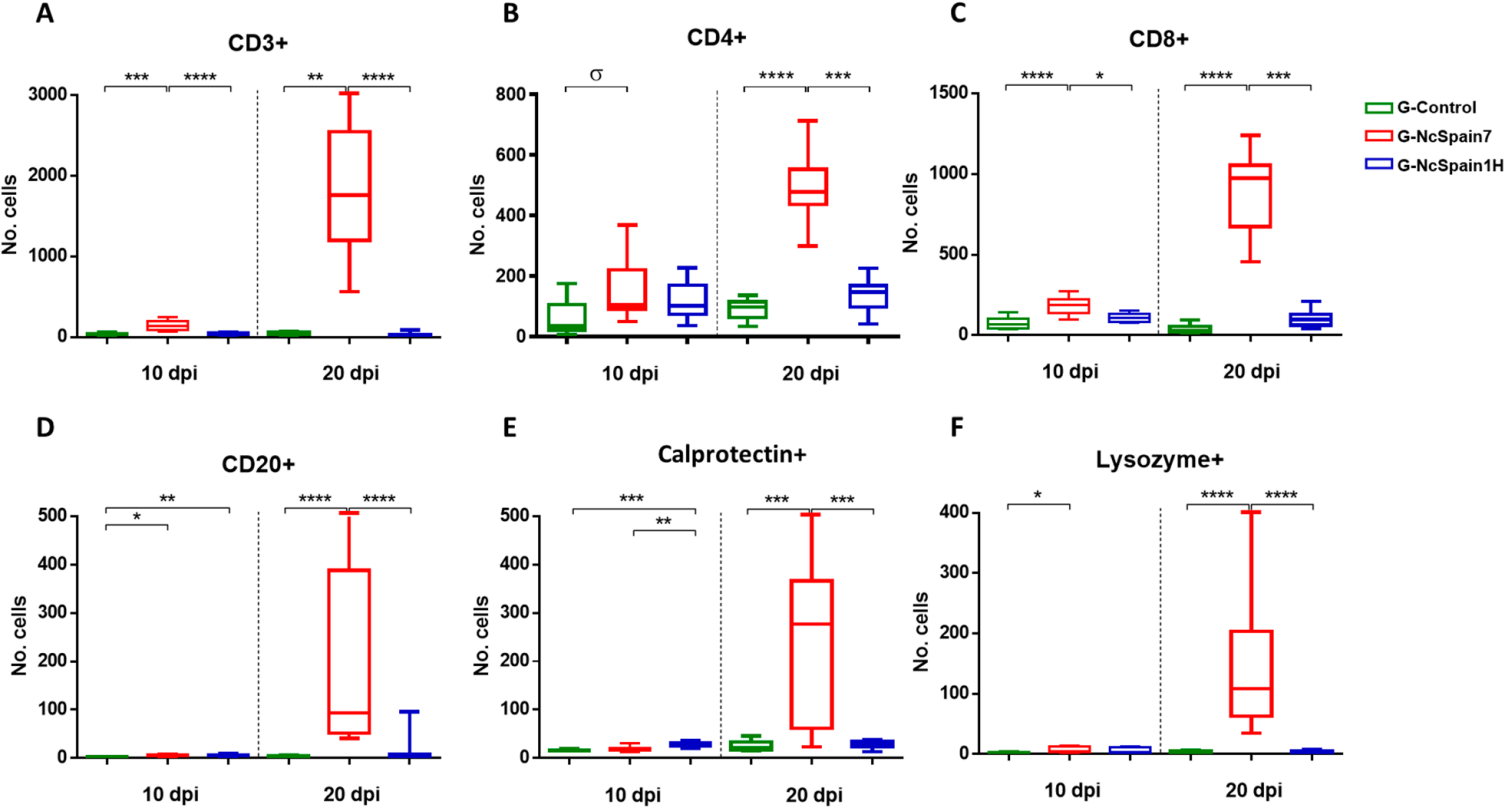
**Quantification of the immunohistochemically labelled T and B lymphocytes and phagocytic cells in the placenta.** Box-plot graphs representing the median number of cells, lower and upper quartiles (boxes) and minimum and maximum values (whiskers) of CD3 + **(A)**, CD4 + **(B)**, CD8 + **(C)**, CD20 + **(D)**, calprotectin + **(E)** and lysozyme + **(F)** cell populations in placentomes collected at 10 and 20 dpi with PBS (G-control) and 10^7^ tachyzoites of Nc-Spain7 (G-NcSpain7) and Nc-Spain1H (G-NcSpain1H) isolates. ****, ***, ** and * indicate *P* < 0.0001, *P* < 0.001, *P* < 0.01 and *P* < 0.05 significant differences, respectively, and σ indicates *P* < 0.1. Box-plot graphs are representative of three randomly selected medial placentomes from each animal, and cells were counted in 20 random 20 × fields of each placentome.

**Figure 6:**
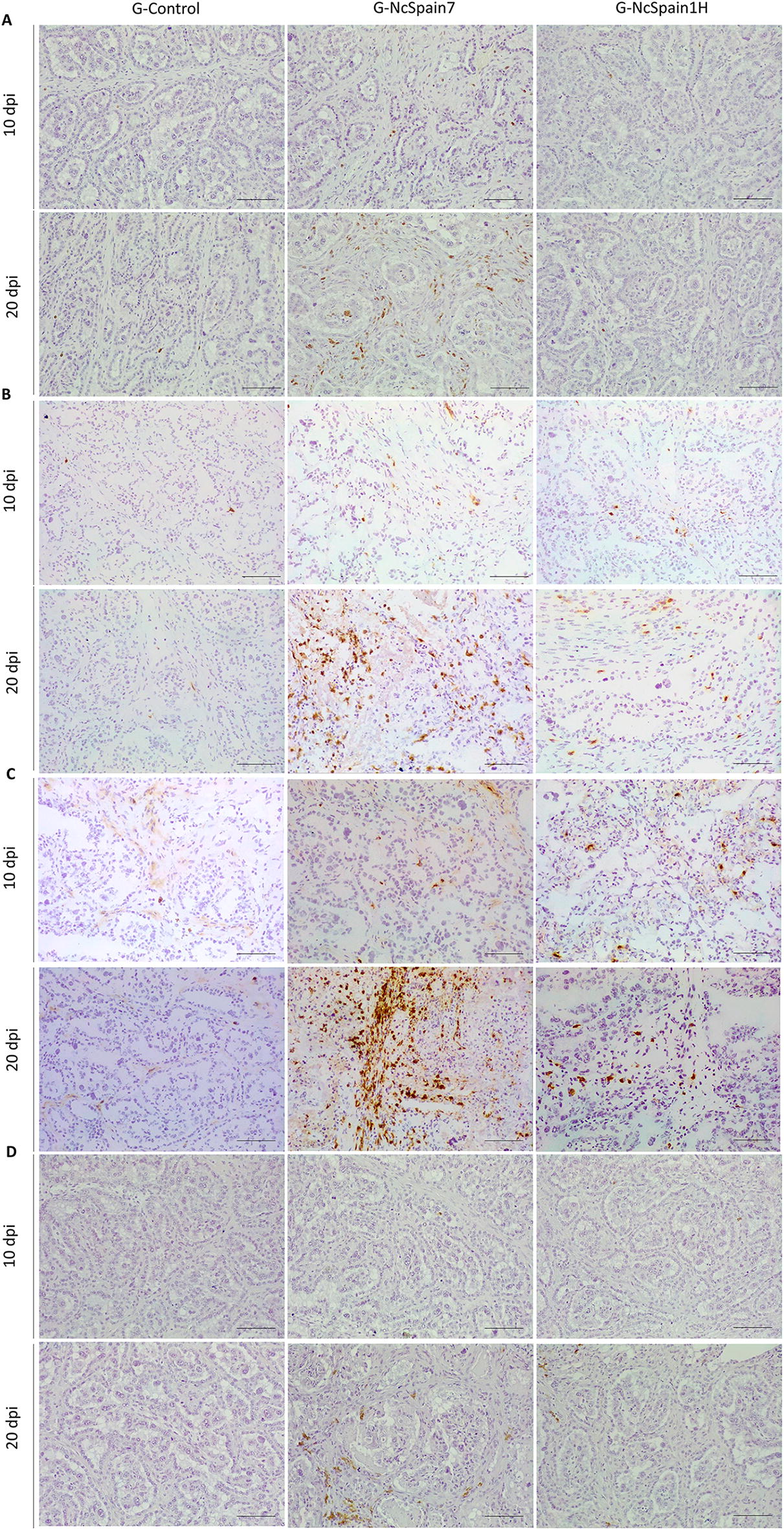
**Comparison of the immunohistochemical labelling of lymphocytes in the placenta**. This panel compares the distribution and frequency of CD3 + **(A)**, CD4 + **(B)**, CD8 + **(C)** and CD20 + **(D)** cells in placentomes from uninfected animals (G-control) and Nc-Spain7- and Nc-Spain1H-infected animals at 10 and 20 dpi. Brownish staining indicates a positive reaction; IHC 10 × , bar 200 µm. Three randomly selected medial placentomes from each animal were analysed.

The staining of the placentomes infected with the highly virulent Nc-Spain7 isolate revealed an increase in the T lymphocyte population (CD3 +) (*P* < 0.01–0.0001; Figure  5A). The distribution of the T cells was diffuse along the interdigitate zone of the placentome, with multifocal infiltrates in relation to lesions that appeared only in Nc-Spain7-infected animals culled at 20 dpi [13] (Figure  6A), associated with the presence of parasite antigen. CD3 + cells were also detected in the caruncular basement and, in small numbers, in the foetal mesenchyme. Nc-Spain1H induced a moderate increase of lymphocyte T population (Figure  5A). Regarding T cell subpopulations, Nc-Spain7 induced the increased infiltration of T CD8 + lymphocytes (*P *< 0.05–0.0001), whereas a significant difference in the number of T CD4 + lymphocytes was not detected at the early stage of infection (Figure  5B, C). Later, at 20 dpi, significant increases in both cell subpopulations were found in Nc-Spain7-infected placentomes (*P* < 0.001–0.0001; Figure  5B, C). In summary, T CD8 + lymphocytes predominated in Nc-Spain7-infected placentas at 10 and 20 dpi (*P* < 0.01–0.001), whereas Nc-Spain1H and uninfected placentas showed a change in the CD4/CD8 ratio at 20 dpi, with T CD4 + lymphocytes predominating over T CD8 + lymphocytes (*P* < 0.01). The distribution of CD4 + and CD8 + cells was diffuse or as sparse aggregates in the caruncular septa at 10 dpi. However, at 20 dpi, and mainly in the group infected with the highly virulent isolate Nc-Spain7, CD4 + and CD8 + cells appeared widely distributed in the placentome, mainly in the caruncular septa, but they were also present in the foetal villi (Figure  6B, C).

Here, we also studied the presence of B lymphocytes by means of CD20 antigen detection. At 10 dpi, the B cell population was significantly increased in caruncular stalks of Nc-Spain7- (*P* < 0.05) and Nc-Spain1H- (*P* < 0.01) infected placentomes, whereas at 20 dpi, the highly virulent isolate induced a marked increase in the B lymphocyte population (*P* < 0.0001; Figure  5D). The CD20 + cells were focally distributed in the maternal stroma next to the necrotic areas (Figure  6D) and diffusely distributed along the caruncular basement membrane.

### Nc-Spain7 infection induced an increase in phagocytic cells in the placenta at 20 dpi that was associated with the presence of lesions

**Figure 7:**
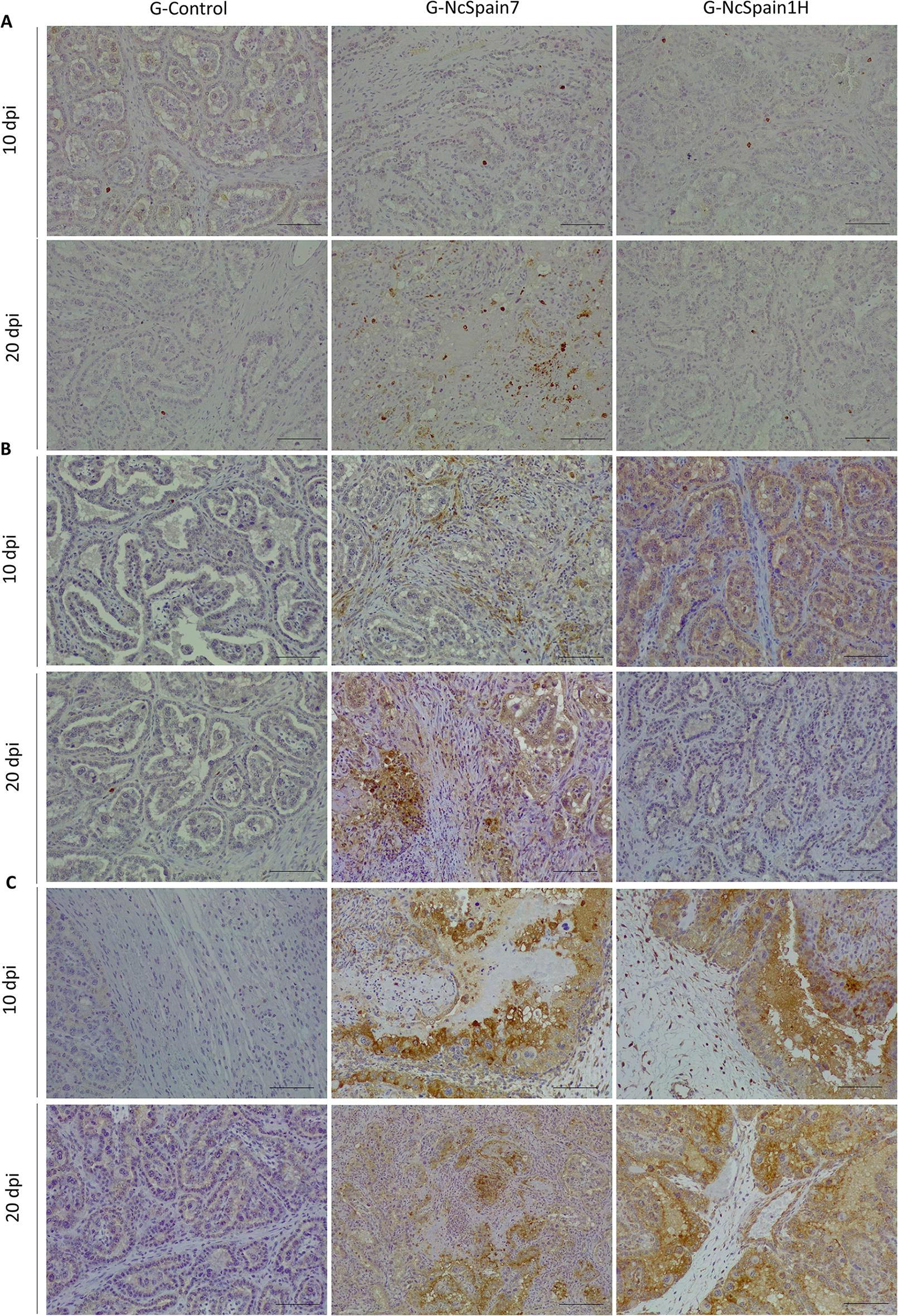
**Comparison of the immunohistochemical labelling of phagocytic cells in the placenta**. This panel compares the distribution and frequency of calprotectin + **(A)**, lysozyme + **(B)** and iNOS + **(C)** cells in placentomes from uninfected animals (G-control) and Nc-Spain7- and Nc-Spain1H-infected animals at 10 and 20 dpi. Brownish staining indicates a positive reaction; IHC 10 × , bar 200 µm. Three randomly selected medial placentomes from each animal were analysed.

At day 10 dpi, Nc-Spain1H infection induced an increase in the number of calprotectin + cells (*P* < 0.01–0.001) that may be associated with the increase in pro-inflammatory cytokine levels (Figure  5E). The number of lysozyme + cells increased slightly after Nc-Spain7 infection (*P* < 0.05; Figure  5F), and iNOS + staining was found in trophoblast cells infected by both isolates. At 20 dpi, calprotectin and lysozyme labelling was increased in placentomes infected with Nc-Spain7 (*P* < 0.001–0.0001; Figure  5E, F), correlating with the striking upregulation of pro-inflammatory cytokine expression (*P* < 0.05). iNOS staining continued to increase in trophoblast cells from both infected groups (Figure  7C), suggesting that NO production may represent a trophoblast-intrinsic defence mechanism against *N. caninum* infection. The distribution of phagocytic cells differed between the early and late stages of infection, being diffuse along the placentome, particularly in the caruncular septa, at 10 dpi and focal, associated with necrotic lesions (Figure  7A–C) and the presence of parasite antigens, at 20 dpi. Notably, iNOS + cells were mainly foetal trophoblasts (Figure  7C).

### Differences in the expression pattern between placentas from live and dead foetuses implicated certain molecules in foetal death

**Figure 8:**
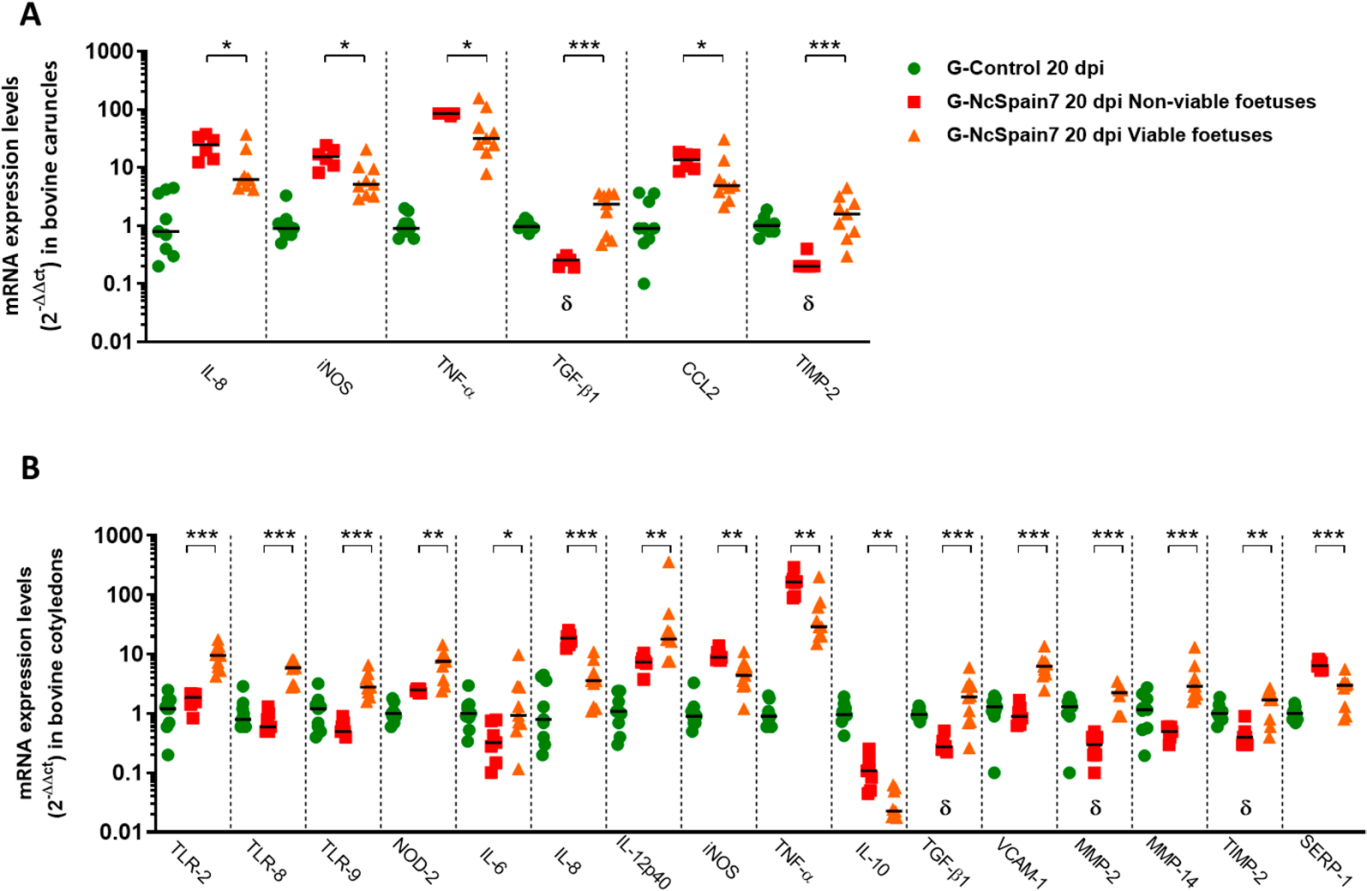
**Comparison of PRRs, cytokine, chemokine, endothelial adhesion molecule, MMP and TIMP transcript expression between animals carrying NVFs and VFs from the G-NcSpain7 group**. Scatter-plot graphs of relative mRNA expression levels (as the fold change) of PRRs, pro- and anti-inflammatory/regulatory cytokines, chemokines, endothelial adhesion molecules, MMPs and TIMPs that were differentially expressed between animals carrying NVFs and VFs in the CA **(A)** and CO **(B)** collected at 20 dpi from cows experimentally infected with Nc-Spain7 at 110 days of gestation. Data are presented as individual points. Horizontal lines represent median values for each group. ****, ***, ** and * indicate *P* < 0.0001, *P* < 0.001, *P* < 0.01 and *P* < 0.05 significant differences, respectively, and σ indicates *P* < 0.1. The δ symbol indicates lower expression compared to that in the uninfected control group. Three randomly selected medial caruncles and cotyledons from each animal were analysed.

**Figure 9:**
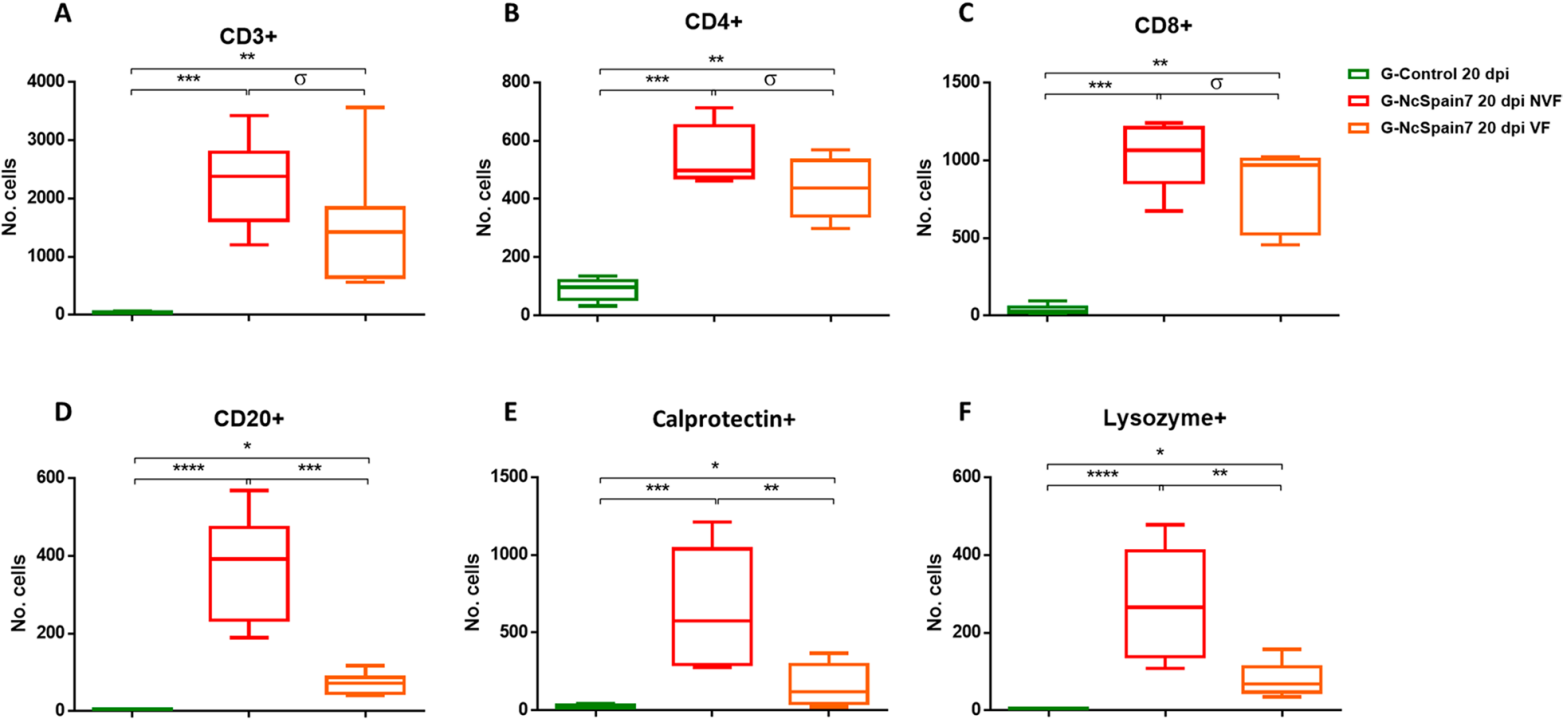
**Comparison of immunohistochemically labelled T and B lymphocytes and macrophages in the placentas of animals carrying NVFs and VFs from the G-NcSpain7 group**. Box-plot graphs representing the median number of cells, lower and upper quartiles (boxes) and minimum and maximum values (whiskers) of CD3 + **(A)**, CD4 + **(B)**, CD8 + **(C)**, CD20 + **(D)**, MAC387 + **(E)** and lysozyme + **(F)** cell populations in placentomes collected at 20 dpi with 10^7^ tachyzoites of Nc-Spain7 at 110 days of gestation. Data from uninfected control placentomes at 20 dpi are also presented. ****, ***, ** and * indicate *P* < 0.0001, *P* < 0.001, *P* < 0.01 and *P* < 0.05 significant differences, respectively, and σ indicates *P* < 0.1. Three randomly selected medial placentomes from each animal were analysed.

Foetal death was found only in animals infected with Nc-Spain7 at 20 dpi (2/5 foetuses). We included mRNA expression and IHC comparisons between animals carrying VFs and NVFs. In the placentome (CA and CO) samples from NVFs at 20 dpi we observed a down-regulated expression of PRRs (TLR-2, TLR-8, TLR-9, and NOD2), cytokines (IL-6, IL-12p40, and TFG-β1), as well as the endothelial adhesion molecule VCAM-1 and ECM regulators (MMP-2, MMP-14 and TIMP-2) compared to FV. However, the pro-inflammatory IL-8, iNOS and TNF-α, the chemokine CCL2 and SERP-1 expression was upregulated in NFVs (Figure  2A, B). In addition, there was diminishing of mRNA MMP-2, TIMP-2 and TFG-β1 levels in animals carrying NVFs (specific *P* values are indicated in Figure  8A, B). The decreased detection of TIMP-2 could be associated with the loss of trophoblast giant cells (TGC) observed in the damaged areas (Figure  2C). Moreover, placentas from NVFs presented an increase in the populations of CD4 + and CD8 + T cells (*P* < 0.1; Figure  9B, C), B lymphocytes (CD20 + ; *P* < 0.001; Figure  9D), macrophages (calprotectin + and lysozyme + , *P* < 0.01), and iNOS + staining; Figure  9E, F).

## Discussion

The placenta is an immunomodulatory organ that can recognize pathogens, culminating in the synthesis of cytokines and recruitment of immune cells to the damaged area, phenomena that may have direct antiparasitic effects or cause immunopathology [2, 4, 5, 15]. Here, we investigated the role of the bovine placenta in the pathogenesis of neosporosis during the initial events of infection (10 and 20 dpi) to identify factors that could explain why some isolates precipitate foetal death (Nc-Spain7) and others are transmitted without causing pathology (Nc-Spain1H). In this study, dramatic differences in the immune responses mounted against each isolate in the placenta were found. Specifically, the low-virulence Nc-Spain1H isolate induced early (10 dpi) expression of genes involved in pathogen recognition, chemokines and pro-inflammatory cytokines at the maternal-foetal interface that remained steady across the studied period to later stages (20 dpi). This result is quite remarkable, since pro-inflammatory cytokines have been implicated in host defence during *N. caninum* infection [1–3, 15, 16], and they lead to enhanced control of Nc-Spain1H growth and dissemination. In addition, in previous in vitro studies, we showed stronger stimulation of the immune response in bovine macrophages [12] and trophoblast cells [9, 11] infected by Nc-Spain1H than in those infected by Nc-Spain7. How placental tissues can recognize and mount an appropriate immune response against this isolate is a fascinating question whose answer remains unknown. Previously, increased expression of genes potentially implicated in the induction of pro-inflammatory immune responses against Nc-Spain1H, such as those coding for SRS proteins (highly immunogenic surface proteins), were observed in bovine target cells [17, 18]. The existence of soluble factors produced by Nc-Spain1H might also contribute to its earlier recognition. These processes need further elucidation.

On the other hand, an increase in local pro-inflammatory cytokines could cause immunopathology, compromising gestation [2, 4, 15, 16]. To prevent tissue damage in the Nc-Spain1H-infected placenta, the pro-inflammatory cytokines are counterbalanced by increased expression of anti-inflammatory and regulatory cytokines (IL-4, IL-10 and TGF-β1). Interestingly, IL-4 and IL-10 expression was higher in the caruncle than in the foetal placenta, suggestive of a mechanism to prevent rejection and preserve gestation. Previously, a down-regulation of Th2 immune responses and regulatory cytokines were observed in *N. caninum*-infected pregnant cattle at 110 days of gestation which had aborted or had non-viable foetuses at euthanasia [19]. The Th2 cytokine profile is favourable for foetal tolerance but also benefits parasite proliferation [2, 15, 16], allowing vertical transmission of Nc-Spain1H. Certainly, in Nc-Spain1H-infected animals, lesions were not found, but parasite DNA was detected in the placenta from one animal at 20 dpi [13]. Nc-Spain1H also seems to induce other mechanisms implicated in lesion resolution. Specifically, higher expression levels of the cytokines IL-17A, TGF-β1 and IL-6 were detected only in Nc-Spain1H placentomes. These cytokines are described to stimulate the release of procollagen and fibronectin [20–22], components of the ECM involved in maintaining placental homeostasis. The transcriptome of a bovine trophoblast cell line infected with Nc-Spain1H showed enrichment of pathways related to ECM organization and extracellular junctions [9]. Here, Nc-Spain1H induced a balance in the expression of MMPs and TIMPs. MMPs modulate cytokine and chemokine activity, and when the infection is cleared, they initiate tissue repair processes [23–25]. However, MMPs degrade ECM components and can lead to immunopathology [25]. MMP activity could be abolished as a result of TIMP expression induced in Nc-Spain1H placentomes, contributing to decreases in ECM remodelling and inflammation, which would result in repair of the lesion area [25]. The increase in the expression of MMP2 and TIMP2 detected by qPCR was not reflected by IHC. Other mechanisms could be involved in the regulation of these genes after transcription or the proteins themselves, such as protein turnover. In addition, in Nc-Spain1H group, minimal variances of ECM immunoreactivity intensity were expected because lesions were not observed and the presence of the parasite was detected in one placenta sample from only one animal at 20 dpi [13]. Collectively, these results suggest an improved adaptation of the low-virulence isolate compared with the high-virulence isolate to replicate in the placenta without causing placental damage, thus promoting ECM remodelling and maintaining a delicate balance between the induction of pro-inflammatory and regulatory immune responses to ensure foetal survival and vertical transmission.

Strikingly, the highly virulent isolate Nc-Spain7 induced a biphasic response in placental tissues. Initially (10 dpi), the parasite seemed to successfully bypass or manipulate the immune system, since the expression of PRRs pro-inflammatory and chemoattractant genes was impaired. Another remarkable result was the complete absence of TLR-9, TLR-8 (in foetal cotyledons), IL-6, TGF-β1 and IL-17A activation by Nc-Spain7, in accordance with the observation that this parasite triggers a downregulated cellular response. Similarly, Nc-Spain7 induced a lower TLR activation and secretion of pro-inflammatory cytokines in a bovine trophoblast cell line [11], suppressed key bovine macrophage functions, such as pathogen killing and pro-inflammatory responses [12], and enhanced expression of genes potentially involved in immune response evasion was observed [18]. Previously, TLR-9 was either not responsive to *N. caninum* in the placenta from heifers experimentally infected with NC-1 isolate on day 70 of gestation [26]. The elucidation of all these mechanisms may provide novel insights into how *N. caninum* evades immune responses while ensuring dissemination and ought to be relevant for bovine vaccination strategies. Despite the lack of activation of classical PRRs at the placental level, the increases in IFN-γ, TNF-α and IL-4, as well as the inflammatory cell infiltration observed at 10 dpi, suggest that other pathways of the immune system could be activated by the highly virulent isolate, initiating intracellular signalling cascades and culminating in the synthesis of these pro-inflammatory cytokines. Effector molecules of the innate immune response, such as NLRs, have been described to be involved in IFN-γ induction during *N. caninum* infection in mice [27]. Recently, NLRs (NAIP, NOD2 and NLRC4) have been implicated in *N. caninum* recognition and macrophage activation [García-Sánchez et al., submitted], but their role needs further investigation.

The evasion of immune responses may facilitate the survival of Nc-Spain7 in its intracellular niche, leading to subsequent multiplication of the parasite in the bovine placenta [10]. At this later stage (20 dpi), the expression of PRRs, pro-inflammatory cytokines and chemokines (especially IFN-γ, TNF-α and IL-12p40) was dramatically enhanced. In a previous study, IFN-γ was the cytokine that showed the highest upregulation at the materno-foetal interface in infected cattle at day 110 of gestation, especially in the caruncle [28]. Although a cytokine storm may be beneficial to eliminate a high parasite load, overexpression of inflammatory mediators may also cause immunopathology [2, 15, 16], contributing to the profound placental damage observed here. In bovine neosporosis, an exacerbated pro-inflammatory response has also been described in placentas from aborted cattle [8, 19, 29, 30]. In line with this observation, we tried to identify key factors involved in abortion by comparing data from placentomes from animals carrying non-viable and viable foetuses. Placentomes from animals carrying non-viable foetuses showed higher parasite burdens, more severe histopathological changes [13] and more extensive inflammatory infiltrates characterized by an increase in the populations of CD4 + and CD8 + T cells. Cantón et al. [31] also suggested a positive association between high number of T lymphocytes (CD3 + , CD4 + , CD8 + and γδ) in the placental infiltrate and occurrence of abortion. The expression of molecules such as IL-8, iNOS and TNF-α, previously associated with placental inflammation, luteolysis and abortion [32, 33], was upregulated in these animals. Interestingly, TGF-β1 expression was diminished, similar to previous in vitro observations [11]. TGF-β1 dysregulation might also contribute to the foetal death observed in Nc-Spain7-infected heifers since it is crucial for neutralizing the Th1 inflammatory response [4]. In addition, a profound alteration in ECM organization was observed, characterized by loss of fibronectin, vimentin and collagen in necrotic foci and downregulation of MMP and TIMP expression. In addition, increased expression of SERP-1, a component of the complement and coagulation cascades [34], was observed in placentomes from animals carrying nonviable foetuses. These mechanisms can play an important role in the pathogenesis of bovine neosporosis and should be investigated in greater depth. Collectively, these findings support the hypothesis that exacerbated immune responses and ECM disruption, together with the high parasite burden and severe lesions found in Nc-Spain7-infected placentas, contributed to foetal death.

Another unknown to be determined is the mechanisms used by the parasite to cross the placental barrier. In vitro infections showed that trophoblasts are the niche for parasite proliferation, whereas caruncular cells seem to restrict the infection, acting as a barrier to parasite transmission [10]. Parallel results in human placentas infected with *Toxoplasma gondii* have also been observed [35]. In the present study, immunohistochemical labelling revealed an increase in the immune cell populations associated with the maternal compartment of the placentome. However, *Neospora* antigen was found only in foetal trophoblast villi cells, confirming that these cells are targets for *N. caninum* proliferation. These results may indicate that the parasite is first recognized in the maternal part of the placenta, subsequently stimulating the maternal immune response and infecting foetal trophoblast villi cells to spread to the foetus. Marin et al. [26] also suggested that the initial recognition of the protozoa at placental level would occur in the maternal fraction. However, the mechanisms by which *N. caninum* (extra- or intra-cellularly) crosses biological barriers are largely unknown. High- and low-virulence isolates may use a combination of different putative complementary pathways. A working hypothesis states that *N. caninum* may exploit immune host cells via a Trojan horse-like mechanism to cross the placental barrier and disseminate [36], similar to *T. gondii* [37]. Accordingly, adoptive transfer of infected dendritic cells in mice promoted *N. caninum* vertical transmission [36], and enhanced hypermotility and transmigration of bovine macrophages was observed [12]. We hypothesized that the Trojan horse-like mechanism could be favoured by Nc-Spain7 because higher invasion and replication rates were observed in bovine macrophages [12], and greater leucocyte infiltration was evidenced in the sites of inflammation during Nc-Spain7 infection than during Nc-Spain1H infection. These leucocytes can be possible targets of parasites that hijack the cell, delivering the parasite to foetal tissues and facilitating parasite dissemination. In addition, the intrinsic invasion and replication abilities of the virulent isolate, combined with the severe lesions and loss of ECM components in the infected placentomes, may benefit the crossing of Nc-Spain7 into the underlying foetal mesenchyme. During Nc-Spain1H infection, ECM modulation may be advantageous for the transmission of immune cells or tachyzoites to the foetus. During *T. gondii* infection, MMPs and TIMPs have been suggested to be involved in crossing of biological barriers [38, 39]. However, the importance of all these mechanisms in the dissemination of *N. caninum* remains unknown.

Our results show that the crosstalk between the host immune response and isolate virulence at the maternal-foetal interface determines the outcome of *Neospora* infection and suggest the existence of different adaptation strategies used by this parasite for transmission to offspring. Infection with the low-virulence isolate leads to the induction of a balanced Th1/Th2 immune response to ensure foetal survival and parasite transmission. Conversely, infection with the high-virulence isolate triggers immune evasion mechanisms that could allow its multiplication during the first cycles of propagation. As an attempt by the host to protect itself, a subsequent exacerbated pro-inflammatory response is produced, leading to profound placental damage and foetal death. This study also provides important information on the immunological and cellular hallmarks of the host-parasite interaction that will facilitate further investigation of the key pathways influencing protection or abortion. Many of these host–pathogen interactions are expected to be governed by parasite effector proteins acting as virulence factors of *N. caninum*, supporting the need for new studies. These investigations will allow the identification of putative vaccine candidates capable of inducing protective immune responses without exacerbating immunopathology.

## Supplementary information

**Additional file 1: Sequences of primers used for cytokine real-time PCR (qPCR) and standard curve data.**

**Additional file 2: Antibodies, specificity and immunohistochemical procedure used.**

**Additional file 3: Th1/Th2 balance in bovine caruncles (A) and cotyledons (B) infected by Nc-Spain1H and Nc-Spain7.** ****, **and *indicate *P* < 0.0001, *P* < 0.01 and *P* < 0.05 significant differences.

## Data Availability

The datasets supporting the conclusions of this article are included within the article and its additional files.

## Abbreviations

CA: Caruncles
CCL: Chemokine (C–C motif) ligand
CO: Cotyledons
Ct: Cycle threshold value
dpi: Days post-infection
ECM: Extracellular matrix
GAPDH: Glyceraldehyde-3-phosphate dehydrogenase
ICAM: Intercellular adhesion molecule
IFN: Interferon
IHC: Immunohistochemistry
IL: Interleukin
iNOS: Inducible nitric oxide synthase
MMP: Metalloproteinase
NLR: NOD-like receptor
NOD: Nucleotide-binding oligomerization domain-like receptors
NVF: Non-viable foetuses
PBS: Phosphate buffer saline
PRRs: Pattern recognition receptors
qPCR: Quantitative real‑time polymerase chain reaction
SERP: Serpine
SRS: SAG1-related sequence
TGF: Transforming growth factor
TIMP: Matrix metalloproteinase inhibitor
TLR: Toll‑like receptor
TNF: Tumoral necrosis factor
VCAM: Vascular cell adhesion molecule
VF: Viable foetuses

## References

[1] Dubey JP, Schares G, Ortega-Mora LM (2007). Epidemiology and control of neosporosis and *Neospora caninum*. Clin Microbiol Rev.

[2] Innes EA (2007). The host-parasite relationship in pregnant cattle infected with *Neospora caninum*. Parasitology.

[3] Dubey JP, Buxton D, Wouda W (2006). Pathogenesis of bovine neosporosis. J Comp Pathol.

[4] Entrican G (2002). Immune regulation during pregnancy and host-pathogen interactions in infectious abortion. J Comp Pathol.

[5] Olmos-Ortiz A, Flores-Espinosa P, Mancilla-Herrera I, Vega-Sánchez R, Díaz L, Zaga-Clavellina V (2019). Innate immune cells and Toll-like receptor-dependent responses at the maternal-fetal interface. Int J Mol Sci.

[6] Rojo-Montejo S, Collantes-Fernández E, Blanco-Murcia J, Rodríguez-Bertos A, Risco-Castillo V, Ortega-Mora LM (2009). Experimental infection with a low virulence isolate of *Neospora caninum* at 70 days gestation in cattle did not result in foetopathy. Vet Res.

[7] Regidor-Cerrillo J, Gómez-Bautista M, Sodupe I, Aduriz G, Álvarez-García G, Del Pozo I, Ortega-Mora LM (2011). In vitro invasion efficiency and intracellular proliferation rate comprise virulence-related phenotypic traits of *Neospora caninum*. Vet Res.

[8] Regidor-Cerrillo J, Arranz-Solís D, Benavides J, Gómez-Bautista M, Castro-Hermida JA, Mezo M, Pérez V, Ortega-Mora LM, González-Warleta M (2014). *Neospora caninum* infection during early pregnancy in cattle: how the isolate influences infection dynamics, clinical outcome and peripheral and local immune responses. Vet Res.

[9] Horcajo P, Jiménez-Pelayo L, García-Sánchez M, Regidor-Cerrillo J, Collantes-Fernández E, Rozas D, Hambruch N, Pfarrer C, Ortega-Mora LM (2017). Transcriptome modulation of bovine trophoblast cells in vitro by *Neospora caninum*. Int J Parasitol.

[10] Jiménez-Pelayo L, García-Sánchez M, Regidor-Cerrillo J, Horcajo P, Collantes-Fernández E, Gómez-Bautista M, Hambruch N, Pfarrer C, Ortega-Mora LM (2017). Differential susceptibility of bovine caruncular and trophoblast cell lines to infection with high and low virulence isolates of *Neospora caninum*. Parasit Vectors.

[11] Jiménez-Pelayo L, García-Sánchez M, Regidor-Cerrillo J, Horcajo P, Collantes-Fernández E, Gómez-Bautista M, Hambruch N, Pfarrer C, Ortega-Mora LM (2019). Immune response profile of caruncular and trophoblast cell lines infected by high- (Nc-Spain7) and low virulence (Nc-Spain1H) isolates of *Neospora caninum*. Parasit Vectors.

[12] García-Sánchez M, Jiménez-Pelayo L, Horcajo P, Regidor-Cerrillo J, Ólafsson EB, Bhandage AK, Barragan A, Werling D, Ortega-Mora LM, Collantes-Fernández E (2019). Differential responses of bovine monocyte-derived macrophages to infection by *Neospora caninum* isolates of high and low virulence. Front Immunol.

[13] Jiménez-Pelayo L, García-Sánchez M, Vázquez P, Regidor-Cerrillo J, Horcajo P, Collantes-Fernández E, Blanco-Murcia J, Gutiérrez-Expósito D, Román-Trufero A, Osoro K, Benavides J, Ortega-Mora LM (2019). Early *Neospora caninum* infection dynamics in cattle after inoculation at mid-gestation with high (Nc-Spain7)-or low (Nc-Spain1H)-virulence isolates. Vet Res.

[14] Schmittgen TD, Livak KJ (2008). Analyzing real-time PCR data by the comparative C(T) method. Nat Protoc.

[15] Quinn HE, Ellis JT, Smith NC (2002). *Neospora caninum*: a cause of immune-mediated failure of pregnancy?. Trends Parasitol.

[16] Almería S, Serrano-Pérez B, López-Gatius F (2017). Immune response in bovine neosporosis: protection or contribution to the pathogenesis of abortion. Microb Pathog.

[17] Horcajo P, Xia D, Randle N, Collantes-Fernández E, Wastling J, Ortega-Mora LM, Regidor-Cerrillo J (2018). Integrative transcriptome and proteome analyses define marked differences between *Neospora caninum* isolates throughout the tachyzoite lytic cycle. J Proteomics.

[18] García-Sánchez M, Jiménez-Pelayo L, Horcajo P, Cerrillo JR, Collantes-Fernández E, Ortega-Mora LM (2019). Gene expression profiling of *Neospora caninum* in bovine macrophages reveals differences between isolates associated with key parasite functions. Front Cell Infect Microbiol.

[19] Almería S, Serrano-Pérez B, Darwich L, Mur-Novales R, García-Ispierto I, Cabezón O, López-Gatius F (2016). Cytokine gene expression in aborting and non-aborting dams and in their foetuses after experimental infection with *Neospora caninum* at 110 days of gestation. Vet Parasitol.

[20] Choy E, Rose-John S (2017). Interleukin-6 as a multifunctional regulator: inflammation, immune response, and fibrosis. J Scler Relat Dis.

[21] Feng JS, Yang Z, Zhu YZ, Liu Z, Guo CC, Zheng XB (2014). Serum IL-17 and IL-6 increased accompany with TGF-beta and IL-13 respectively in ulcerative colitis patients. Int J Clin Exp Med.

[22] Dufour AM, Alvarez M, Russo B, Chizzolini C (2018). Interleukin-6 and type-I collagen production by systemic sclerosis fibroblasts are differentially regulated by interleukin-17A in the presence of transforming growth factor-beta 1. Front Immunol.

[23] Geurts N, Opdenakker G, Van den Steen PE (2012). Matrix metalloproteinases as therapeutic targets in protozoan parasitic infections. Pharmacol Ther.

[24] Korpos E, Wu C, Song J, Hallmann R, Sorokin L (2010). Role of the extracellular matrix in lymphocyte migration. Cell Tissue Res.

[25] Parks WC, Wilson CL, López-Boado YS (2004). Matrix metalloproteinases as modulators of inflammation and innate immunity. Nat Rev Immunol.

[26] Marin MS, Hecker YP, Quintana S, Pérez SE, Leunda MR, Cantón GJ, Cobo ER, Moore DP, Odeón AC (2017). Toll-like receptors 3, 7 and 8 are upregulated in the placental caruncle and fetal spleen of *Neospora caninum* experimentally infected cattle. Vet Parasitol.

[27] Wang X, Gong P, Zhang X, Li S, Lu X, Zhao C, Yu Q, Wei Z, Yang Y, Liu Q, Yang Z, Li J, Zhang X (2018). NLRP3 inflammasome participates in host response to *Neospora caninum* infection. Front Immunol.

[28] Almería S, Araujo RN, Darwich L, Dubey JP, Gasbarre LC (2011). Cytokine gene expression at the materno-foetal interface after experimental *Neospora caninum* infection of heifers at 110 days of gestation. Parasite Immunol.

[29] Rosbottom A, Gibney EH, Guy CS, Kipar A, Smith RF, Kaiser P, Trees AJ, Williams DJ (2008). Upregulation of cytokines is detected in the placentas of cattle infected with *Neospora caninum* and is more marked early in gestation when fetal death is observed. Infect Immun.

[30] Maley SW, Buxton D, Macaldowie CN, Anderson IE, Wright SE, Bartley PM, Esteban-Redondo I, Hamilton CM, Storset AK, Innes EA (2006). Characterization of the immune response in the placenta of cattle experimentally infected with *Neospora caninum* in early gestation. J Comp Pathol.

[31] Cantón GJ, Katzer F, Maley SW, Bartley PM, Benavides-Silván J, Palarea-Albaladejo J, Pang Y, Smith SH, Rocchi M, Buxton D, Innes EA, Chianini F (2014). Inflammatory infiltration into placentas of *Neospora caninum* challenged cattle correlates with clinical outcome of pregnancy. Vet Res.

[32] Clark DA, Chaouat G, Arck PC, Mittruecker HW, Levy GA (1998). Cytokine-dependent abortion in CBA x DBA/2 mice is mediated by the procoagulant fgl2 prothrombinase. J Immunol.

[33] Liu H, Liu Z, Chao H, Li Z, Song Z, Yang Y, Peng JP (2014). High-dose interferon-γ promotes abortion in mice by suppressing Treg and Th17 polarization. J Interferon Cytokine Res.

[34] Chen H, Davids JA, Zheng D, Bryant M, Bot I, van Berckel TJ, Biessen E, Pepine C, Ryman K, Progulski-Fox A, Kesavalu L, Moyer R, McFadden G, Lucas A (2013). The serpin solution; targeting thrombotic and thrombolytic serine proteases in inflammation. Cardiovasc Hematol Disord: Drug Targets.

[35] Ander S, Rudzki E, Arora N, Sadovsky Y, Coyne CB, Boyle JP (2018). Human placental syncytiotrophoblasts restrict *Toxoplasma gondii* vertical transmission at two distinct stages and induce CCL22 in response to infection. mBio.

[36] Collantes-Fernandez E, Arrighi RB, Alvarez-García G, Weidner JM, Regidor-Cerrillo J, Boothroyd JC, Ortega-Mora LM, Barragan A (2012). Infected dendritic cells facilitate systemic dissemination and transplacental passage of the obligate intracellular parasite *Neospora caninum* in mice. PLoS One.

[37] Lambert H, Vutova PP, Adams WC, Loré K, Barragan A (2009). The T*oxoplasma gondii*-shuttling function of dendritic cells is linked to the parasite genotype. Infect Immun.

[38] Wang M, Lai S (2013). Fibronectin degradation by MMP-2/MMP-9 in the serum of pregnant women and umbilical cord with *Toxoplasma gondii* infection. J Obstet Gynaecol.

[39] Ólafsson EB, Varas-Godoy M, Barragan A (2018). *Toxoplasma gondii* infection shifts dendritic cells into an amoeboid rapid migration mode encompassing podosome dissolution, secretion of TIMP-1, and reduced proteolysis of extracellular matrix. Cell Microbiol.

[40] Menzies M, Ingham A (2006). Identification and expression of Toll-like receptors 1–10 in selected bovine and ovine tissues. Vet Immunol Immunopathol.

[41] Deng Z, Shahid M, Zhang L, Gao J, Gu X, Zhang S, Zou J, Fanning S, Han B (2016). An investigation of the innate immune response in bovine mammary epithelial cells challenged by *Prototheca zopfii*. Mycopathologia.

[42] Magee DA, Taraktsoglou M, Killick KE, Nalpas NC, Browne JA, Park SD, Conlon KM, Lynn DJ, Hokamp K, Gordon SV, Gormley E (2012). Global gene expression and systems biology analysis of bovine monocyte-derived macrophages in response to in vitro challenge with *Mycobacterium bovis*. PLoS One.

[43] Arranz-Solís D, Benavides J, Regidor-Cerrillo J, Horcajo P, Castaño P, del Carmen Ferreras M, Jiménez-Pelayo L, Collantes-Fernández E, Ferre I, Hemphill A, Pérez V (2016). Systemic and local immune responses in sheep after *Neospora caninum* experimental infection at early, mid and late gestation. Vet Res.

[44] Sakumoto R, Hayashi KG, Fujii S, Kanahara H, Hosoe M, Furusawa T, Kizaki K (2017). Possible roles of CC-and CXC-chemokines in regulating bovine endometrial function during early pregnancy. Int J Mol Sci.

[45] Merriman KE, Powell JL, Santos JE, Nelson CD (2018). Intramammary 25-hydroxyvitamin D3 treatment modulates innate immune responses to endotoxin-induced mastitis. J Dairy Sci.

[46] Peralta MB, Baravalle ME, Belotti EM, Stassi AF, Salvetti NR, Ortega HH, Rey F, Velázquez MM (2017). Involvement of matrix metalloproteinases and their inhibitors in bovine cystic ovarian disease. J Comp Pathol.

[47] Dilly M, Hambruch N, Shenavai S, Schuler G, Froehlich R, Haeger J, Ozalp G, Pfarrer C (2011). Expression of matrix metalloproteinase (MMP)-2, MMP-14 and tissue inhibitor of matrix metalloproteinase (TIMP)-2 during bovine placentation and at term with or without placental retention. Theriogenology.

[48] Puech C, Dedieu L, Chantal I, Rodrigues V (2015). Design and evaluation of a unique SYBR Green real-time RT-PCR assay for quantification of five major cytokines in cattle, sheep and goats. BMC Vet Res.

[49] NCBI National Center for Biotechnology Information. https://www.ncbi.nlm.nih.gov/. Accessed 20 November 2018

