# Supplementary material for: Crosstalk between Neospora caninum and the bovine host at the maternal-foetal interface determines the outcome of infection
Source: Vet Res. 2020 Jun 17;51:83. doi: 10.1186/s13567-020-00803-y (PMC7302351; doi:10.1186/s13567-020-00803-y)
Supplement: Supplementary file 1 — Additional file 1: Sequences of primers used for cytokine real-time PCR (qPCR) and standard curve data. [file 13567_2020_803_MOESM1_ESM.docx]

**Additional file 1 Sequences of primers used for cytokine real-time PCR (qPCR) and standard curve data.**

| **Target^a^** | **Primer** | **Primer sequences (5’-3’)** | **Product size (bp)** | ***R*^2 b^** | **Slope^c^** | **First description** |
| --- | --- | --- | --- | --- | --- | --- |
| TLR-2 (NM_001048231.1) | QTLR2-UP | ACGACGCCTTTGTGTCCTAC | 192 | 0.993 | (-3.74) – (-3.38) | [40] |
|  | QTLR2-RP | CCGAAAGCACAAAGATGGTT |  |  |  |  |
| TLR-3 (NM_001008664.1) | TLR-3-F | GAGGCAGGTGTCCTTGAACT | 329 | 0.995 | (-3.52) – (-3.32) | [40] |
|  | TLR-3-R | GCTGAATTTCTGGACCCAAG |  |  |  |  |
| TLR-8 (AY957629.1) | TLR-8-F | GACGCTTCTGTCACGGACTG | 152 | 0.994 | (-3.624) – (-3.381) | This study |
|  | TLR-8-R | TTGCTTTGGTTGATGCTCTG |  |  |  |  |
| TLR-9 (AY957630.1) | TLR-9-F | AGTTCCCTCGTGTCCCTGT | 216 | 0.996 | (-3.771) – (-3.582) | This study |
|  | TLR-9-R | CACCTCCGTGAGGTTGTTGT |  |  |  |  |
| NOD-2 (NM_001002889.1) | qNOD2-F | AGACCACCACGGATATGTACC | 238 | 0.996 | (-3.595) – (-3.568) | [41] |
|  | qNOD2-R | AGCACCAGGAAGCCAAGAG |  |  |  |  |
| IL-1β (NM_174093.1) | IL-1β-F | ACCTGAACCCATCAACGAAATG | 74 | 0.99 | (-3.586) – (-3.450) | [42] |
|  | IL-1β-R | TAGGGTCATCAGCCTCAAATAACA |  |  |  |  |
| IL-6  (X68723.1) | QIL-6-UP | CTGGGTTCAATCAGGCGATT | 150 | 0.999 | (-3.22) – (-3.20) | [43] |
|  | QIL-6-RP | GGATCTGGATCAGTGTTCTGA |  |  |  |  |
| IL-8  (BC103310.1) | qIL8-Fw | CCACACCTTTCCACCCCAAA | 177 | 0.995 | (-3.36) – (-3.23) | [11] |
|  | qIL8-Rw | CTTGCTTCTCAGCTCTCTTC |  |  |  |  |
| IL-12p40 (NM_174356.1) | QIL12-UP | AGTACACAGTGGAGTGTCAG | 157 | 0.992 | (-3.39) – (-3.35) | [8] |
|  | QIL12-RP | TTCTTGGGTGGGTCTGGTTT |  |  |  |  |
| IL-17A (NM_001008412.1) | qIL17-F | CACAGCATGTGAGGGTCAAC | 83 | 0.998 | (-3.454) – (-3.369) | [10] |
|  | qIL17-R | GGTGGAGCGCTTGTGATAAT |  |  |  |  |
| iNOS (NM_001076799.1) | qiNOS-F2 | CGAACCTGCAGGTCTTTGAC | 152 | 0.997 | (-3.644) – (-3.638) | This study |
|  | qiNOS-R2 | GAAGTCATGCTTCCCATCGC |  |  |  |  |
| IFN-γ (NM_174086.1) | QIFN-UP | GATTCAAATTCCGGTGGATG | 110 | 0.994 | (-3.47) – (-3.30) | [8] |
|  | QIFN-RP | TTCTCTTCCGCTTTCTGAGG |  |  |  |  |
| TNF-α (EU276079.1) | QTNF-UP | CCAGAGGGAAGAGCAGTCC | 126 | 0.998 | (-3.39) – (-3.27) | [8] |
|  | QTNF-RP | GGAGAGTTGATGTCGGCTAC |  |  |  |  |
| IL-4  (M77120.1) | QIL4-UP | CTGCCCCAAAGAACACAACT | 169 | 0.995 | (-3.33) – (-3.54) | [8] |
|  | QIL4-RP | GTGCTCGTCTTGGCTTCATT |  |  |  |  |
| IL-10 (NM_174088.1) | QIL10-UP | TGCTGGATGACTTTAAGGGTTACC | 60 | 0.999 | (-3.27) – (-3.42) | [8] |
|  | QIL10-RP | AAAACTGGATCATTTCCGACAAG |  |  |  |  |
| TGF-β1 (NM_001009400.1) | QTGF-UP | GGTGGAATACGGCAACAAAA | 117 | 0.999 | (-3.60) – (-3.53) | [43] |
|  | QTGF-RP | CGAGAGAGCAACACAGGTTC |  |  |  |  |
| CCL2 (MCP-1)(NM_174006.2) | CCL2-F | TGCAGACCCCAAGCAGAAAT | 144 | 0.997 | (-3.461) – (-3.388) | [44] |
|  | CCL2-R | AGAGGGCAGTTAGGGAAAGC |  |  |  |  |
| CCL4 (NM_001075147.2) | CCL4-F | AGCTGTGGTATTCCAGACCAA | 87 | 0.998 | (-3.526) – (-3.184) | [42] |
|  | CCL4-R | TCAAGGTCATCCACGTACTCC |  |  |  |  |
| CCL5 (RANTES) (NM_175827) | qCCL5-F | CACCCACGTCCAGGAGTATT | 116 | 0.998 | (-3.480) – (-3.345) | [45] |
|  | qCCL5-R | CTCGCACCCACTTCTTCTCT |  |  |  |  |
| ICAM-1 (NM_174348.2) | qICAM-Fw | AGACCTATGTCCTGCCATCG | 219 | 0.994 | (-3.34) – (-3.30) | [11] |
|  | qICAM-Rw | GGTGCCCTCCTCATTTTCCT |  |  |  |  |
| VCAM-1 (XM_005204079.2) | qVCAM-Fw | GAACTGGAAGTCTACATCTC | 128 | 0.998 | (-3.36) – (-3.32) | [11] |
|  | qVCAM-Rw | CAGAGAATCCGTGGAGCTGG |  |  |  |  |
| MMP-2 (NM_174745.2) | qMMP2-F | TCTTCGCCGGAGACAAATTCTGGA | 133 | 0.998 | (-3.671) – (-3.646) | [46] |
|  | qMMP2-R | ATCCAGGTTATCAGGGATGGCGTT |  |  |  |  |
| MMP13 (NM_174389.2) | MMP13-F | GGAACTAAAGAGCACGGTGAC | 162 | 0.999 | (-3.427) – (-3.372) | [9] |
|  | MMP13-R | GGCAGCGACAAGAAACAAG |  |  |  |  |
| MMP-14 (AF144758.1) | MMP14-F | ACTTGGAAGGGGGACACC | 235 | 0.998 | (-3.543) – (-3.310) | [47] |
|  | MMP14-R | AGGGGGCATCTTAGTGGG |  |  |  |  |
| TIMP-1 (NM_174471.3) | TIMP-1-F | TCGTGGGGACCGCAGAAGT | 134 | 0.992 | (-3.354) – (-3.297) | [46] |
|  | TIMP-1-R | CTCCATGGCAGGGGTGTAGAT |  |  |  |  |
| TIMP-2 (NM_174472.3) | qTIMP2-F2 | CCCATCAAGCGGATTCAGTAT | 136 | 0.999 | (-3.656) – (-3.593) | [46] |
|  | qTIMP2-R2 | ACTCCTTCTTTCCTCCAATGTC |  |  |  |  |
| SERP-1 (NM_174137.2) | qSERP1-F2 | GACCCTTCACCAAAGATGAG | 137 | 0.995 | (-3.305) – (3.184) | This study |
|  | qSERP1-R3 | CTGCGAAATTCAGGATGCGGA |  |  |  |  |
| GAPDH (NM_001034034) | GAPDH-F | ATCTCGCTCCTGGAAGATG | 227 | 0.996 | (-3.67) – (3.58) | [48] |
|  | GAPDH-R | TCGGAGTGAACGGATTCG |  |  |  |  |
| β- ACTIN (NM_173979.3) | BACTIN-UP | ACACCGCAACCAGTTCGCCAT | 216 | 0.994 | (-3.45) – (-3.36) | [8] |
|  | BACT216-RP | GTCAGGATGCCTCTCTTGCT |  |  |  |  |

^a^ NCBI accession numbers are for cDNA sequences used in primer design. Primer annealing was also checked with the *Bos taurus* genomic DNA sequences [49].

^b^ Minimum coefficient of regression (*R^2^*) of standard curves for each PCR target in all batches of amplification.

^c^ Standard curve slopes. Minimum and maximum values for slopes for each PCR target in all batches of amplification.
