# Supplementary material for: Crosstalk between Neospora caninum and the bovine host at the maternal-foetal interface determines the outcome of infection
Source: Vet Res. 2020 Jun 17;51:83. doi: 10.1186/s13567-020-00803-y (PMC7302351; doi:10.1186/s13567-020-00803-y)
Supplement: Supplementary file 2 — Additional file 2: Antibodies, specificity and immunohistochemical procedure used. [file 13567_2020_803_MOESM2_ESM.docx]

**Additional file 2** **Antibodies, specificity and immunohistochemical procedure used.**

| **Antigen** | **Clone** | **Type** | **Marker for** | **Tissue** | **Antigen retrieval** | **Dilution** | **Source** |
| --- | --- | --- | --- | --- | --- | --- | --- |
| ***N. caninum*** | – | Rabbit,  Policlonal | *N. caninum* antigens | Paraffin embedded | Trypsin | 1:1000 | Saluvet group |
| **CD3** | – | Rabbit,  Policlonal | T cells | Paraffin embedded | Heat and pressure; citrate buffer, pH6 | 1:300 | Dako, Denmark |
| **CD4** | CC30 | Mouse, Monoclonal | T helper cells | Frozen | None | 1:100 | Serotec, USA |
| **CD8** | CC63 | Mouse, Monoclonal | Cytotoxic T cells | Frozen | None | 1:50 | Serotec, USA |
| **CD20** | – | Rabbit,  Policlonal | B cells | Paraffin embedded | None | 1:200 | Thermo Fisher Scientific, USA |
| **Calprotectine** | MAC387 | Mouse, Monoclonal | Macrophages and activated epithelial cells | Paraffin embedded | 96 °C 20’buffer Dako pH9 | 1:200 | GeneTex, USA |
| **Lysozyme** | – | Rabbit,  Policlonal | Lysozyme | Paraffin embedded | 96 °C 20’buffer Dako pH6 | 1:250 | Dako, Denmark |
| **iNOS** | – | Rabbit,  Policlonal | Inducible nitric oxid synthetasa | Paraffin embedded | 96 °C 20’buffer Dako pH9 | 1:100 | Novus, USA |
| **MMP-2** | – | Rabbit,  Policlonal | Matrix metalloproteinase 2 | Paraffin embedded | None | 1:75 | Thermo Fisher Scientific, USA |
| **MMP-14** | Ab-2 | Rabbit,  Policlonal | Matrix metalloproteinase 14 | Paraffin embedded | None | 1:150 | Thermo Fisher Scientific, USA |
| **TIMP2** | 67-4H11 | Mouse, Monoclonal | Metallopeptidase inhibitor 2 | Paraffin embedded | None | 1:2500 | Chemicon, USA |
| **Fibronectin** | IST-3 | Mouse, Monoclonal | Fibronectin | Paraffin embedded | 96 °C 20’citrate buffer pH6 | 1:200 | Sigma-Aldrich, USA |
| **Vimentin** | Vim 3B4 | Mouse, Monoclonal | Vimentin | Paraffin embedded | 96 °C 20’citrate buffer pH6 | 1:100 | Dako, Denmark |
| **Collagen Type IV** | – | Rabbit, Policlonal | Collagen Type IV | Paraffin embedded | 96 °C 20’citrate buffer pH6 | 1:400 | Acris-antibodies, Germany |
