# Supplementary figures and images for: Crosstalk between Neospora caninum and the bovine host at the maternal-foetal interface determines the outcome of infection
Source: Vet Res. 2020 Jun 17;51:83. doi: 10.1186/s13567-020-00803-y (PMC7302351; doi:10.1186/s13567-020-00803-y)

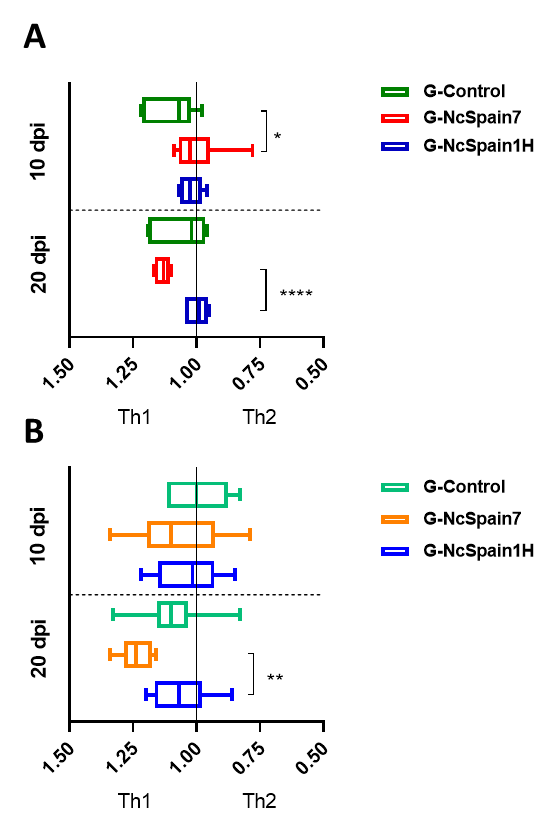

Supplement: Supplementary file 3 — Additional file 3: Th1/Th2 balance in bovine caruncles (A) and cotyledons (B) infected by Nc-Spain1H and Nc-Spain7. ****, **and *indicate P < 0.0001, P < 0.01 and P < 0.05 significant differences. [file 13567_2020_803_MOESM3_ESM.docx]
